# The TGF‐b/SOX4 axis and ROS‐driven autophagy co‐mediate CD39 expression in regulatory T‐cells

**DOI:** 10.1096/fj.201902664

**Published:** 2020-04-22

**Authors:** Marlene C. Gerner, Liesa S. Ziegler, Ralf L. J. Schmidt, Martin Krenn, Fritz Zimprich, Keziban Uyanik‐Ünal, Vassiliki Konstantopoulou, Sophia Derdak, Giorgia Del Favero, Ilse Schwarzinger, Kaan Boztug, Klaus G. Schmetterer

**Affiliations:** ^1^ Department of Laboratory Medicine Medical University of Vienna Vienna Austria; ^2^ Department of Neurology Medical University of Vienna Vienna Austria; ^3^ Department of Surgery Medical University of Vienna Vienna Austria; ^4^ Department of Pediatrics Medical University of Vienna Vienna Austria; ^5^ Core Facility Genomics Medical University of Vienna Vienna Austria; ^6^ Department of Food Chemistry and Toxicology Faculty of Chemistry University of Vienna Vienna Austria; ^7^ Research Center for Molecular Medicine of the Austrian Academy of Sciences Vienna Austria

**Keywords:** autophagy, CD39, regulatory T‐cells, immune tolerance, SOX4, TGF‐b signaling

## Abstract

The ectonucleotidase CD39 on human regulatory T‐cells (Treg) is an important immune regulator which is dysregulated in autoimmune diseases and cancer immunosuppression. We here define that CD39 expression on Treg is independent of the Treg‐specific transcription factors FOXP3 and HELIOS and promoted by canonical TGF‐b‐ and mTOR‐signaling. Furthermore, the TGF‐b mediated upregulation of CD39 is counteracted by reactive oxygen species (ROS)‐driven autophagy. In line, CD39^+^ peripheral blood Treg constitute a distinct lineage with low autophagic flux and absent ROS production. Patients with rare genetic defects in autophagy show supraphysiological levels of CD39^+^ Treg, validating our observations in vivo. These biological processes rely on a distinct transcriptional program with CD39^+^ Treg expressing low levels of two genes with putative involvement in autophagy, *NEFL* and *PLAC8*. Furthermore, the TGF‐b downstream transcription factor *SOX4* is selectively upregulated in CD39^+^ Treg. Overexpression of *SOX4* in Treg strongly increases CD39 expression, while Crispr/Cas9‐mediated knockout of *SOX4* in Treg has the opposing effect. Thus, we identify a crucial role of SOX4 in immune regulation and provide new insights involving the interplay of tolerogenic cues and autophagy in Treg.

**Abbreviations**
AntA: Antimycin A
atRA: all trans retinoic acid
BafA: Bafilomycin A1
BHT: Butylated hydroxytoluene
CQ: chloroquine
HC: calcium hydroxycitrate
iTreg: in vitro induced Treg
NAC: N‐Acetyl‐Cysteine
RAPA: rapamycin
ROS: reactive oxygen species
Treg: regulatory T‐cells
tTreg: thymus‐derived Treg

## 1 INTRODUCTION

The immune system involves highly regulated processes with activating and suppressive signals keeping each other in balance. This guarantees an appropriate immune response against pathogens while preventing overshooting or misdirected reactions. Among the mechanisms of immune tolerance, regulatory T‐cells (Treg) play a crucial role. In vivo, Treg arise as a distinct CD4^+^ T‐cell lineage in the thymus during T‐cell maturation (thymus‐derived Treg; tTreg) or can be induced from naïve T‐cells in the periphery under tolerogenic conditions (peripherally induced Treg; pTreg).^1^ Similarly, naïve T‐cells can be polarized toward Treg in vitro (induced Treg; iTreg) by culture with suppressive signals such as all trans retinoic acid (atRA) and TGF‐b^2^ or the immunosuppressive drug rapamycin (RAPA).^3^ The master transcription factor FOXP3 governs Treg phenotype and function.^4^, ^5^ However, recent studies have suggested that FOXP3‐independent mechanisms exist in Treg.^6^ In this regard, the importance of other transcription factors such as HELIOS^7^, ^8^ or RUNX1^9^, ^10^ has been demonstrated.

Treg utilize multiple different molecular mechanisms to suppress activation of immune cells.^11^ Among others, expression of CD39, an ectonucleotidase that degrades extracellular ATP and ADP to AMP, was defined as an important factor for the regulatory capacity of Treg.^12^, ^13^ The suppressive potency of CD39 is thus two‐fold. First, extracellular proinflammatory ATP,^14^, ^15^ is removed. Furthermore, the concerted action of CD39 and a second ectoenzyme, CD73, leads to the generation of extracellular adenosine, which inhibits effector T‐cell function via binding to the A2A‐R.^16^ Recent reports have also described that CD39 inactivates isoprenoid‐derived Vγ9Vδ2 T‐cell phosphoantigens, adding a third immunosuppressive function.^17^

In murine T‐cells, CD39 is uniformly expressed on tTreg in a Foxp3‐dependent manner and can be used as a reliable surface marker for these cells similar to CD25. Furthermore, CD39 is crucially involved in their suppressive function in vitro and in vivo.^12^, ^13^ In contrast to murine tTreg, human tTreg do not uniformly express CD39.^13^ Recent studies have defined that expression of CD39 segregates human tTreg into two subpopulations which show a high interindividual variability.^13^, ^18^, ^19^ These two subsets are also functionally distinct, as CD39^+^ tTreg display higher suppressive capacity, especially regarding the suppression of IFN‐g and IL‐17 production by effector T‐cells.^18^ It is still unclear, how CD39 is regulated on human tTreg. While the CD39 expression status on tTreg is dependent on the genetic background of the individual,^18^ the actual factors regulating CD39 expression in Treg have not been defined. Moreover, CD39 is expressed on a small subset of CD4^+^ as well as CD8^+^ effector T‐cells and is upregulated following activation, showing that CD39 expression on T‐cells is not restricted to Treg.^20^

CD39 function is critically involved in various human pathologies. Reduced CD39 expression has been described in autoimmune diseases including SLE,^21^ inflammatory bowel disease^22^ and multiple sclerosis.^13^ On the contrary, CD39 expression is increased on tumor‐infiltrating Treg in many oncological settings, including solid malignancies such as colon carcinoma,^23^ gastric cancer,^24^ head and neck cancer^25^ as well as hematological malignancies^26^, ^27^, ^28^ and is associated with poor prognosis and lower overall survival. Therefore, CD39 is considered as a relevant immune checkpoint in tumor immunology.^29^, ^30^, ^31^ Thus, the knowledge about mechanisms regulating CD39 expression could open novel therapeutic strategies in several fields of clinical immunology.

Macroautophagy (commonly referred to as autophagy) is a central metabolic process involved in providing energy during stress responses and the degradation of dysfunctional organelles. Designated proteins or organelles are targeted to cytoplasmic vesicles, so called autophagosomes. Subsequently, autophagosomes are fused with lysosomes, where the cargo is degraded. While autophagy is thus pivotal for energy supply and cell homeostasis, the autophagy status also feeds back into many important cellular processes such as differentiation and survival.^32^, ^33^ In this context, a growing number of studies have described links between autophagy and the development and function of immune cells.^34^, ^35^ Recent studies have also defined a role for autophagy in the regulation of the ATP‐CD39 axis in murine tumor models. Knockout of autophagy genes as well as in vivo application of caloric restriction mimetics led to decreased CD39 levels on tumor cells accompanied by increased extracellular ATP and improved antitumor immunity.^36^, ^37^ Thus, strong evidence exists about the involvement of autophagy in the regulation of CD39 expression.

Consequently, in this study, we addressed the functional interplay of tolerogenic signals, ROS production and autophagy in in vitro induced Treg and peripheral blood tTreg from healthy donors in the regulation of CD39. Furthermore, we also analyzed peripheral blood from patients suffering from rare genetic defects resulting in decreased autophagy to gain further insights in an in vivo setting. Finally, transcriptomic comparison of CD39^−^/CD39^+^ tTreg were performed to identify potential genetic mechanisms and transcription factors underlying these cellular processes.

## 2 MATERIALS AND METHODS

### 2.1 Cells and cell culture

Peripheral blood samples of healthy donors were provided by the Austrian Red Cross (Vienna, Austria) upon informed written consent. Peripheral blood mononuclear cells (PBMC) were isolated by standard Ficoll‐Paque centrifugation. CD4^+^ T‐cells were isolated using MagniSort Human CD4 T‐cell Enrichment Kit (Invitrogen/Thermo Fisher Scientific, Waltham, USA) according to manufacturing instructions. Purity was assessed by flow cytometric analyses and found to be above 95% for CD4^+^ T‐cells. All functional assays were performed in IMDM (Gibco, Thermo Fisher Scientific) supplemented with 10% of fetal calf serum (Gibco), 10 µg/mL of gentamycin (Gibco), and 1.25 µg/mL of amphotericin B (Lonza, Walkersville, MD, USA).

### 2.2 Patients and sex and age‐matched controls

The study was approved by the local Ethics Committee of the Medical University of Vienna (EC number EK 1150/2015). Healthy donors (20 female: age range 25‐35 and 20 male: age range 26‐37) were recruited upon informed written consent. A 26 year old male patient with Danon Disease was recruited at the Department of Surgery. Following cardiac transplantation in 2005 due to hypertrophic cardiomyopathy, an immunosuppressive regimen with tacrolimus and mycophenolic acid was initiated. In 2015, the patient developed a posttransplant non‐Hodgkin lymphoma which was successfully treated with Rituximab. At the time of blood draw for this study, the patient was in complete remission. A female patient with polyglucosan body myopathy was recruited at the Department of Neurology, Medical University of Vienna. Clinical features, the genetic defect in the *RBCK‐1* gene as well as basic immunological parameters have been described by us before.^38^

### 2.3 Flow cytometry

For flow cytometric analysis cells were washed in PBS (Gibco) + 0.5% FCS (Gibco) + 0.05% Sodium azide (Sigma Aldrich, St. Louis, MO). Following staining with eFluor450, eFluor506, FITC, PE, APC, PerCP Cy5.5, PE‐Cy7‐, or APC‐Cy7‐conjugated human mononuclear antibodies against CD3 (SK7), CD4 (OKT4, SK3 or RPA‐T4), CD25 (BC96), CD39 (eBioA1), CD45RO (UCHL1), CD73 (AD2), CD127 (eBioRDR5), Helios (22F6), FOXP3 (PCH101), and mouse isotype controls (P3.6.2.8.1 or eBMG2b; all from Invitrogen) and CD25 (2A3, BV711) and CD45RO (UCHL1, BV605; both Becton Dickinson, BD, Franklin Lakes, NJ), cells were incubated at +4°C for 30 minutes and washed once more. For intracellular staining, cells were fixed and permeabilized using eBioscience Foxp3/ Transcription Factor Staining Buffer Set (Invitrogen) according to manufacturing instructions. Cells were analyzed on a FACSCanto II cytometer (BD). For cell‐sorting, cells were washed in PBS + 0.5% FCS + 2 mM EDTA (Sigma Aldrich) and cells were FACS‐sorted on a FACSAria Fusion cell sorter (BD). Naïve CD4^+^ T‐cells were identified by the CD4^+^CD25^−^CD39^−^CD45RO^−^ phenotype. tTregs were identified by the CD4^+^CD25^+^CD127^low^ phenotype and were sorted into CD39^+^ and CD39^−^. Purity was assessed by flow cytometric analyses and found to be above 98%. Flow cytometry data were analyzed using the FlowJo software (version 10, Tree Star, Ashland OR, USA).

### 2.4 Retroviral overexpression of *Helios*, *FOXP3,* and *SOX4*

#### 2.4.1 Molecular cloning

The cDNA encoding human Helios was amplified from cDNA of anti‐CD3/CD28 stimulated PBMC, using the following primers: Helios for 5′‐GCGCCCGAATTCGCCACCATGGAAACAGAGGCTATTGATGGCTATATAACG‐3′, Helios rev 5′‐CCCGCGGCGGCCGCTTTAGTGGAATGTGTGCTCCCCTCGAAC‐3′ (underlined sequences mark restriction enzyme sites). The cDNA was cloned into the pMMP‐IRES‐GFP vector using the restriction enzymes EcoRI and NotI (both Thermo Fisher Scientific). The pMMP‐FOXP3‐IRES‐GFP and the empty‐control pMMP‐IRES‐GFP vector were described elsewhere.^39^ SOX4 was cloned from the pINDUCER21‐SOX4 plasmid into the pMMP‐IRES‐GFP vector. pINDUCER21‐SOX4 was a gift from George Daley (Addgene plasmid # 51304; http://n2t.net/addgene:51304; RRID:Addgene_51304).^40^

#### 2.4.2 Transfection of HEK‐293 cells

HEK‐293 cells were transfected using the Ca_2_PO_4_ precipitation method as described previously.^41^ In short, for the production of amphotropic T‐cell transducing retrovirus supernatants, 30 µg of the pMD‐MoMLV gag‐pol, the envelope encoding pMD‐GalV, and transgene cDNA in the pMMP‐IRES‐GFP vector were diluted in 900 µL of ddH2O and 100 µL of 2.5 M CaCl_2_ were added and incubated for 5 minutes. Afterward, 1 mL 2xHBSS (Sigma Aldrich) was added and the mixture was spread on 293 cells at 10% confluency. On the next day, the mixture was removed and 12 mL fresh medium was added, and cells were cultured for 2 days two allow virus accumulation in the supernatant.

#### 2.4.3 Retroviral transduction

T‐cells (1 × 10^7^/well) were stimulated in 6‐well flat bottom plates with 5 × 10^6^ anti‐CD3/CD28 coated microbeads (beads: Dynabeads, Invitrogen, Carlsbad, CA; Anti‐CD28; clone CD28.2, BD and Muronomab‐CD3, Janssen‐Cilag, Neuss, Germany, clone: OKT3) and 300 U/mL IL‐2 (PeproTech, London, UK) for 48 hours. tTreg (1 × 10^5^/well) were stimulated in 96‐well flat bottoms with 0.5 × 10^5^ anti‐CD3/CD28 coated microbeads and 300 U/mL IL‐2. Retroviral transduction was performed by addition of cell‐free retroviral supernatant in the presence of 8 µg/mL polybrene (Sigma Aldrich) followed by centrifugation at 900 *g* for 90 minutes. Twenty‐four hours after transduction, cells were transferred to fresh medium containing 100 U/mL IL‐2.

### 2.5 In vitro Treg‐induction

Either 1 × 10^6^ or 1 × 10^5^ naïve CD4^+^ T‐cells were preincubated for 1 hour with 100 U/mL IL‐2 + 100 nM all trans retinoic acid (atRA, Sigma Aldrich) + 5 ng/mL TGF‐b (PeproTech) or with 100 U/mL IL‐2 + 100 nM Rapamycin (RAPA, Sigma Aldrich) following anti‐CD3/CD28‐stimulation (ratio cells:beads 2:1). Control cells were cultured in 100 U/mL IL‐2 only. After 4 days in culture, medium + supplements were replaced and after 7 days cells were washed, preincubated in medium + supplements again and were restimulated for another 7 days. After 7 and 14 days of Treg‐induction, cells were stained with monoclonal antibodies against CD25, CD39, CD127, and FOXP3 to identify Treg‐phenotype.

### 2.6 ATP degradation assay

CD25^high^ iTregs or control cells were FACS‐sorted and 7 × 10^4^ cells were cultured in presence of 5 µM ATP in a total volume of 200 µL at 37°C. After 15 and 30 minutes, supernatants were collected and cells were removed by centrifugation. ATP concentration was measured by a luciferase‐based assay using the ATP determination Kit (Invitrogen) according to the manufacturing constructions. Luminescence was detected on a microplate reader (BioTek Instruments, Winooski, VT, USA).

### 2.7 Reagents for TGF‐b‐signaling‐ and mTOR‐inhibition

In some cultures, inhibitors to the canonical TGF‐b signaling factor SMAD3 (5 µM, SIS3, merck Millipore, Darmstadt, Germany) and noncanonical TGF‐b signaling factors p38 (1 µM, SB 203580) and Rock1 (100 nM, GSK429286A; both Selleck Chemicals, Houston, TX) were added during iTreg induction with IL‐2+atRA/TGF‐b. In some cultures, the mTOR inhibitor RAPA (100 nM) was added to the IL‐2+atRA/TGF‐b‐mediated Treg‐induction.

### 2.8 Reagents for autophagy‐modulation and ROS‐modulation

To test the effect of the presence of autophagy‐regulators and ROS‐regulators during Treg‐induction, following compounds were added to the IL‐2/atRA/TGF‐b‐mediated Treg‐induction: autophagy‐inhibitors: chloroquine (CQ) (5 µM), Bafilomycin A1 (1 nM), Ammonium chloride (NH_4_Cl, 1 mM). Autophagy‐inducers: RAPA (100 nM), Calcium hydroxycitrate (1 mM, HC). ROS‐inhibitors: N‐acetyl‐cysteine (1 mM, NAC), Butylated hydroxytoluene (100 µM, BHT). ROS‐inducers: Antimycin A (5 ng/mL, AntA; all Sigma Aldrich). All supplements were added to the medium during preincubation of the cells before activation.

### 2.9 Western blotting

Cells were lysed with RIPA buffer supplemented with protease inhibitor and phosphatase inhibitors (all Sigma Aldrich). After 30 minutes incubation on ice with periodic pulse vortexing, cell‐lysates were centrifuged at 16 000 *g* for 15 minutes at +4°C. For an equal loading, protein concentration was measured using Pierce BCA Protein Assay Kit (Thermo Fisher Scientific) according to manufacturing instructions. A 4%‐12% SDS‐PAGE (Bio‐Rad, Hercules, CA, USA) was used to separate proteins following a transfer onto PVDF membranes (GE Healthcare). The membrane was blocked with bovine serum albumin; BSA (Sigma Aldrich) and the following antibodies were used for incubation over night: phospho‐Smad3 (1:2000, Anti‐Smad3 PhosphoS423+S425, Abcam, Cambridge, UK), total Smad2/3 (1:1000 R&D Systems), LC3 A/B (1:1000 D3U4C, Cell Signaling Technology, Massachusetts, USA), and Pan‐Actin (1:2000, D18C11, Cell Signaling Technology). Protein bands were visualized using HRP‐linked anti‐rabbit (Cell Signaling Technology) or anti‐goat (Abcam) and SuperSignal West Pico Chemiluminescent Substrate (Thermo Fisher Scientific).

### 2.10 Reactive oxygen species (ROS)‐assay

For ROS detection, the DCFDA Cellular ROS Detection Assay Kit (Abcam) was used. In this assay, DCFDA (2′,7′‐dichlorofluorescin diacetate), a fluorogenic dye, is deacetylated by cellular esterases and later oxidized by ROS into 2′,7′‐dichlorofluorescein (DCF), a highly fluorescent compound which correlates with intracellular ROS‐activity and can be detected in the FITC‐channel. Twenty‐four hours after T‐cell activation, 1 × 10^5^ cells were stained in culture medium with 20 μM DCFDA for 30 minutes at 37°C and were then immediately transferred on ice. Without washing, ROS levels were quantified by flow cytometry in the FITC‐channel.

### 2.11 Autophagy Cyto‐ID staining

For measuring autophagic flux in live cells by flow cytometry, the Cyto‐ID Autophagy Detection Kit 2.0 (Enzo Life Sciences, Farmingdale, NY, USA) was used. The Cyto‐ID selectively stains autophagic compartments including pre‐autophagosomes, autophagosomes, and autophagolysosomes. Twenty‐four hours or 72 hours after T‐cell activation, 1 × 10^5^ cells were washed in PBS following by staining with Cyto‐ID (1:1000) for 30 minutes at 37°C. After staining, cells were washed and Cyto‐ID intensity was detected by flow cytometry in the FITC‐channel.

### 2.12 RT‐PCR

At the indicated time points, RNA was isolated by RNeasy Mini Kit or RNeasy Micro Kit (both Qiagen, Hilden, Germany) and genomic DNA was digested by DNase I (Sigma Aldrich). cDNA was generated by random hexamer‐primed reverse transcription (Invitrogen by Thermo Fisher Scientific). Relative transcriptional levels of the indicated genes were quantified using the Luna Universal qPCR Master Mix (New England Biolabs, Ipswich, MA, USA) on a 7900HT Fast Real‐Time PCR system (Applied Biosystems, Foster City, CA, USA). Transcriptional levels of beta‐2‐microglobulin (b2m) or GAPDH were used as reference. Primer sequences are shown in Table S1. For quantification ΔCT values from the respective samples were calculated (ΔCT = CT*_gene_* ‐CT*_GAPDH or b2m_*) and fold‐expression was calculated according to the formula 2^‐(ΔCTSample ‐ΔCTunstimulated). All values were normalized to the fold‐expression of the iTreg control cultures in IL‐2.

### 2.13 Immunofluorescence microscopy

For the immunofluorescence experiments cells were stained as previously described.^42^ Briefly, isolated CD39^−^ and CD39^+^ tTregs were fixed in 3.7% of formaldehyde and permeabilized with Triton X (0.2%) for 10 minutes. Unspecific binding sites were masked with BSA (1% 1 hour) and LC3B was stained using a rabbit polyclonal anti LC3B antibody (dilution 1:500; 2 hours). Afterward, cells were rinsed three times with washing buffer (0.01% Triton in PBS) and donkey anti rabbit IgG Alexa Fluor 568 was used as secondary antibody (depicted in green Figure 5C). At the end of the incubation cells were washed additionally three times with washing buffer and three times with PBS. At the end of the staining, cells were transferred in multichambered microscopy slides (Ibidi GmbH, Martinsried, Germany) and mounted with Roti‐Mount FluoCare (Roth, Graz, Austria) with DAPI to counterstain cell nuclei. If not otherwise specified reagents were from Invitrogen by Thermo Fisher Scientific. Images were acquired with a laser scanning confocal microscope LSM Zeiss 710 equipped with ELYRA PS.1 system with a Plan Apochromat 100X/1.46 oil objective and an AndoriXon 897 (EMCCD) camera.

### 2.14 Transcriptomic analyses

CD39^−^/CD39^+^ tTreg from peripheral blood CD4^+^ T‐cells from three healthy donors were stringently isolated by FACS‐sorting (see above). Total RNA from 3 × 10^5^ was isolated from the respective specimen directly after sorting using the RNeasy Micro Kit (Qiagen). Subsequently, 500pg of total RNA were amplified and labeled using the Affymetrix 3` IVT Pico Kit (Affymetrix Biosystems by Thermo Fisher Scientific) and subjected to microarray analyses using the Affymetrix GeneChip Human PrimeView arrays (Thermo Fisher Scientific) according to the manufacturers' protocol. Signal scanning was performed on an Affymetrix GeneChip Scanner 3000 7G, software AGCC 3.1.1. Affymetrix PrimeView microarray data were processed in R with the affy package,^43^ and differential gene expression was analysed in R with the limma package.^44^, ^45^ Heatmaps were generated in R with coolmap from the limma package, and volcano plots were generated with the EnhancedVolcano package.^46^ Gene set enrichment analysis was performed with GSEA version 3.0^47^ using the Hallmark gene sets from MSigDB^48^ version 6.2.

### 2.15 Crispr/Cas9 knockout

Knockout of SOX4 in transgenic T‐cells or peripheral blood tTreg was performed according to an adapted protocol by Roth et al.^49^ In brief, 1 × 10^6^ cells were preactivated for 2 days with anti‐CD3/CD28‐stimulation (ratio cells:beads 2:1) + rec. human IL‐2 (300 U/mL). RNPs were preassembled in vitro by complexing a two‐component gRNA to Cas9. In brief, lyophilized crRNAs and tracrRNAs (Integrated DNA Technologies, Coralville, IA) were resuspended in TE buffer. A combination of six crRNAs targeting different sequences within the *SOX4* cDNA was used and mixed 1:1 with the tracrRNA and incubated at 95°C for 5 minutes to obtain an 80 µM gRNA. Subsequently, high fidelity recombinant Cas9 (60 µM; IDT) was complexed with the gRNA to generate an RNP complex (final concentration 6 µM). Cells were harvested and activation beads were removed using a magnetic device, washed in PBS and resuspended in Lonza electroporation buffer P3 (Lonza, Basel, Switzerland) according to the manufacturers' protocol. The cells were mixed with the RNP to a total volume of 20 µL and electroporated on a Lonza Nucleofector 4D X unit electroporator (protocol EH 115). Immediately after electroporation, fresh medium was added to the electroporation cuvette and cells were rested for 15 minutes at 37°C. Afterward, cells were cultured for another 7 days in IMDM + 100 U/mL rec. IL‐2 and reactivated using anti‐CD3/CD28 microbeads (beads:cell = 1:1) for another 3‐5 days. Knockout efficiency was determined by Sanger sequencing and analysis using the SYNTHEGO ICE analysis tool and was routinely found >75%. RNA sequences and sequencing primer are shown in Table S2.

### 2.16 Statistical analyses

If not otherwise described, data are represented as mean ± SD. Differences between groups were assed using one‐way ANOVA or paired *t* test, dependent on the number of samples. For multiple‐comparison, Tukey's multiple comparison test or Dunnett's multiple comparison test was used. To test data for normally distribution, KS normality test or D'Agostino & Pearson omnibus normality test was used (dependent on the number of samples). Significance was defined as: not significant (n.s.) *P* > .05; *P ≤ .05; **P < .01; ***P < .001. Data were analyzed using GraphPad Prism (version 6, GraphPad Software, Inc La Jolla, CA, USA).

## 3 RESULTS

### 3.1 Expression of CD39 on human CD4^+^ T‐cells is independent of FOXP3 and HELIOS

**FIGURE 1:**
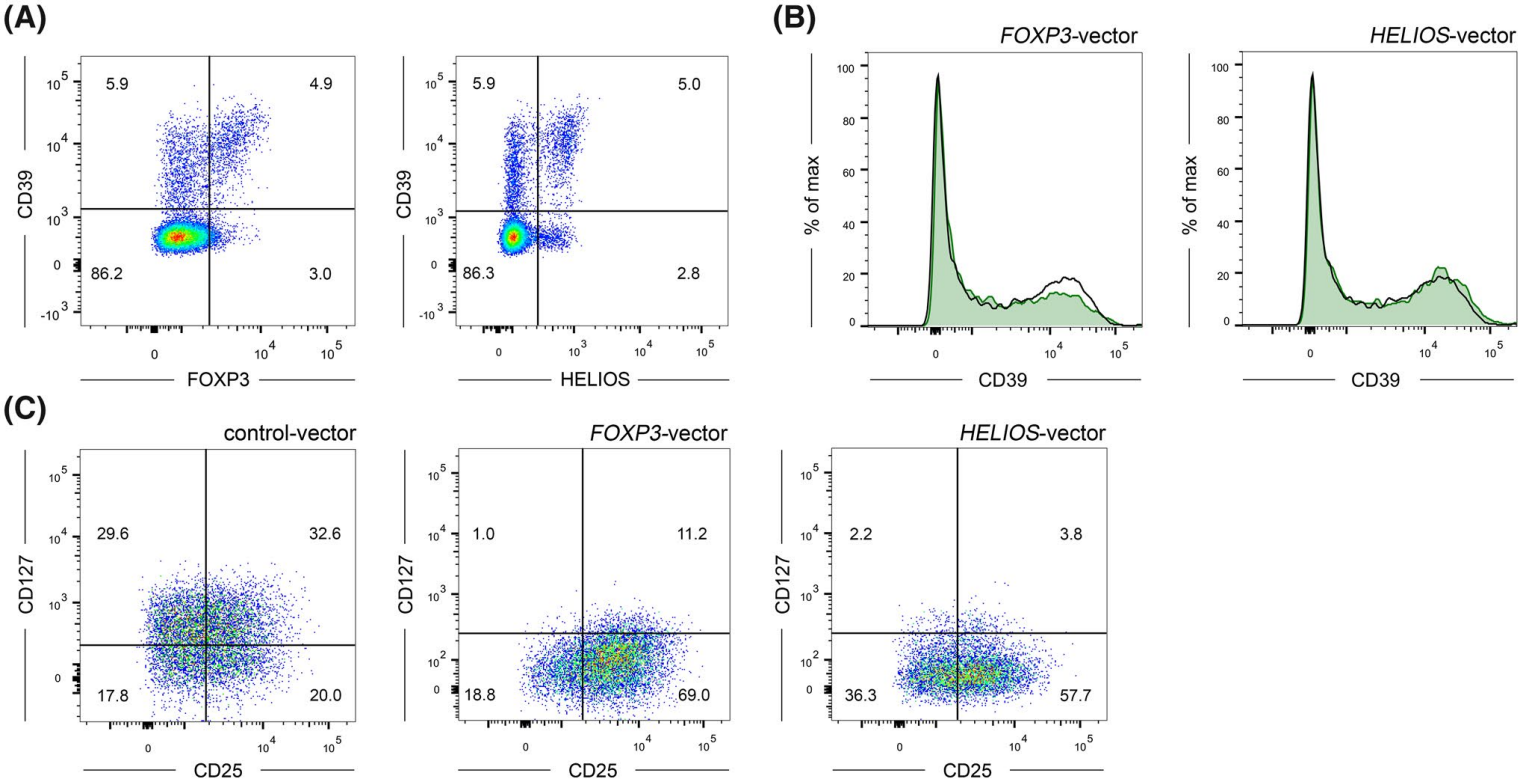
Expression of CD39 on human CD4^+^ T‐cells is independent of FOXP3 and HELIOS. A, Representative FACS‐plots of CD39 (surface) and FOXP3 or HELIOS expression (intracellular) in peripheral blood CD4^+^ T‐cells from one healthy donor (n = 5). B, CD39 expression on CD4^+^GFP^+^ T‐cells following retroviral transduction of the transcription factors FOXP3 (left) or HELIOS (right) using the pMMP‐IRES‐GFP vector. The black line indicates empty vector transduced cells (control), green histograms represent FOXP3 or HELIOS‐transduced cells. C, Expression of the Treg‐related surface markers CD25 and CD127 on CD4^+^GFP^+^ T‐cells. Analyses in (B) and (C) were performed 7 days after transduction

Recently it has been established that CD39 expression on human tTreg is highly variable between individuals and CD39^−^ and CD39^+^ tTreg populations exist.^18^ In accordance, we found that human peripheral blood tTreg, as defined by the CD3^+^CD4^+^CD25^high^CD127^low^ phenotype, showed a high variability in CD39 expression with a range of 6%‐62% CD39^+^ cells in our cohort (see below Figure 7). Furthermore and in accordance with previous reports, CD39 expression was detected on up to 4% of CD3^+^CD4^+^CD25^low^CD127^high^ effector T‐cells (Teff) which do not express FOXP3.^50^ Thus, we hypothesized that CD39 expression on CD4^+^ T‐cells might be independent of FOXP3. Along those lines, also CD4^+^FOXP3^+^ T‐cells were segregated into a CD39^−^ and CD39^+^ subpopulation (Figure 1A). Similarly, no correlation between the Treg‐associated transcription factor HELIOS and CD39 was observed (Figure 1A). Overexpression of the human *FOXP3* and HELIOS cDNA in human CD4^+^CD25^low^CD39^−^ T‐cells did not lead to upregulation of CD39 expression (Figure 1B). However, in accordance with previous studies,^39^ FOXP3 and HELIOS strongly modulated the expression levels of well‐ defined tTreg markers such as CD25 and CD127 (Figure 1C). Thus, the present data demonstrate that expression of CD39 on T‐cells is largely independent of the transcription factors FOXP3 and HELIOS. Surface expression of CD39 was also correlated with surface expression of the AMP‐degrading ectonucleotidase CD73.^13^ We found a clear‐cut segregation of CD39^+^ and CD73^+^ T‐cells with only a negligible percentage of CD39^+^CD73^+^ cells both in the CD4^+^ and the CD8^+^ T‐cell population. CD73 expression in total CD4^+^ T‐cells denoted a distinct subset of about 3%‐16% of all cells, however only minimal expression of CD73 was measured on the surface of tTreg. In contrast, CD8^+^ T‐cells displayed robust CD73 expression with about 21%‐70% positive cells within our donor population (Figure S1).

### 3.2 Expression of CD39 during in vitro Treg‐induction from naïve T‐cells is dependent on atRA/TGF‐b and mTOR signaling

**FIGURE 2:**
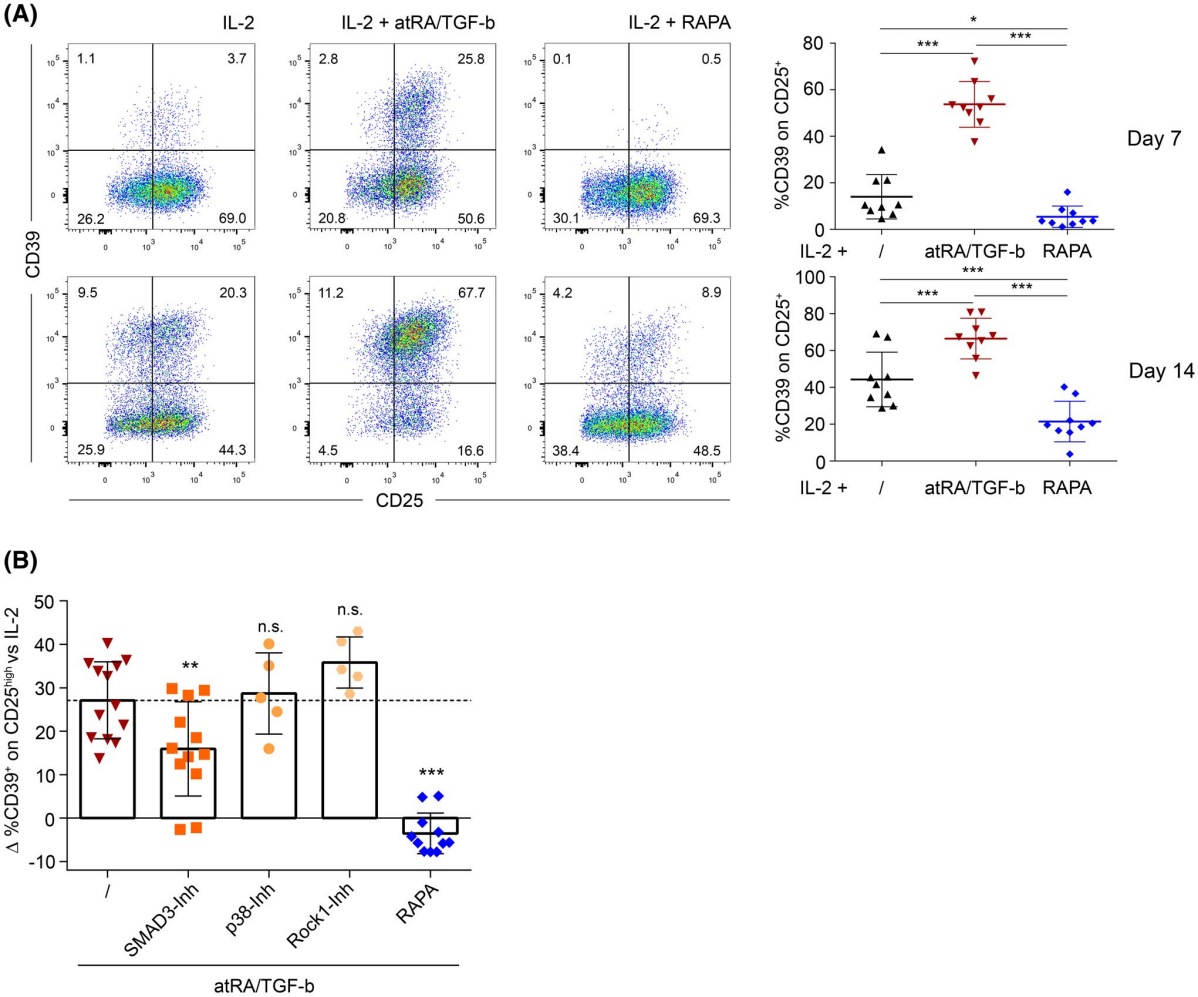
Expression of CD39 is upregulated by atRA/TGF‐b and downregulated by RAPA during in vitro polarization of inducible Treg from naïve T‐cells. A, For iTreg induction, naïve CD4^+^CD25^−^CD39^−^CD45RO^−^ cells were preincubated with IL‐2 in combination with either atRA/TGF‐b or RAPA and cells were stimulated with anti‐CD3/CD28 coated microbeads. Cultures with IL‐2 only served as controls. After 7 and 14 days, surface‐expression of CD25 and CD39 was measured by FACS. Left: representative FACS‐plots from one healthy donor. Right: statistical analysis of CD39^+^ cells within the CD25^+^ population, data are represented as mean ± SD *P ≤ .05; **P < .01; ***P < .001 (one‐way ANOVA). B, Inhibitors of the canonical TGF‐b signaling (SMAD3), noncanonical TGF‐b signaling (p38 and Rock1), and mTOR signaling (RAPA) were added during iTreg induction with IL‐2+atRA/TGF‐b. After 7 days in culture, surface expression of CD39 on CD25^+^ iTreg was measured and CD39 expression values were corrected against values from the control culture. The data are represented as mean ± SD **P < .01; ***P < .001; not significant (n.s.) *P* > .05 (paired *t* test)

Given the independence of CD39 expression from the transcription factors FOXP3 and HELIOS, we aimed to define factors governing CD39 expression on Treg. As a model system, we used the in vitro polarization of naïve CD4^+^CD25^−^CD39^−^ CD45RO^−^ T‐cells into iTreg by two‐week culture in the presence of atRA and TGF‐b^2^ or in the presence of the immunosuppressive drug RAPA.^3^ Cultures with IL‐2 only served as controls. Already after the first week a clear‐cut segregation of CD39 expression between the differentially polarized types of iTreg was observed. While cells cultured with atRA/TGF‐b showed a robust upregulation of CD39 expression, RAPA significantly downregulated induction of CD39 (Figure 2A). This effect became even more pronounced after 2 weeks in culture (Figure 2A). At this time point also about 40% of T‐cells in the control cultures were CD39^+^, indicating that CD39 expression is a default process during activation to a certain extent (Figure 2A). Similar to the situation in peripheral blood tTreg, CD39 expression in all three groups showed a high inter‐donor variability irrespective of the protocol (Figure 2A). In accordance with CD39 expression levels, atRA/TGF‐b iTreg showed the highest ATP degradation followed by cells from the control culture, while RAPA iTreg only showed minimal ATP degradation capacity (Figure S2A). Importantly, neither protocol led to surface expression of CD73 on the iTreg (Figure S2B). For validation of the Treg‐induction protocols, we also compared the suppressive potential of the different iTreg in cocultures with responder T‐cells (Tresp). Both types of iTreg showed robust suppressive capacity toward CD4^+^ and CD8^+^ Tresp (Figure S2C) and were equally effective in the suppression of CD4^+^ Tresp proliferation. In contrast, atRA/TGF‐b iTreg were slightly superior to RAPA iTreg in the suppression of CD8^+^ Tresp proliferation (Figure S2C).

To further define the underlying mechanisms of CD39 upregulation by TGF‐b, we used well‐defined small molecule inhibitors targeting canonical and noncanonical TGF‐b signaling. Only inhibition of the canonical TGF‐b signaling factor SMAD3^51^ led to downregulation of CD39 induction, while inhibition of noncanonical signaling factors (p38 or Rock1)^51^, ^52^ did not affect this process (Figure 2B). Given the opposing roles of atRA/TGF‐b and RAPA, we also combined these substances during iTreg generation. Under these conditions, we found a massive downregulation of CD39 even below the level in IL‐2 control cultures, indicating that mTOR signaling and associated processes are pivotal for the upregulation of CD39 by TGF‐b (Figure 2B).

### 3.3 Autophagy and ROS levels modulate CD39 upregulation by atRA/TGF‐b

**FIGURE 3:**
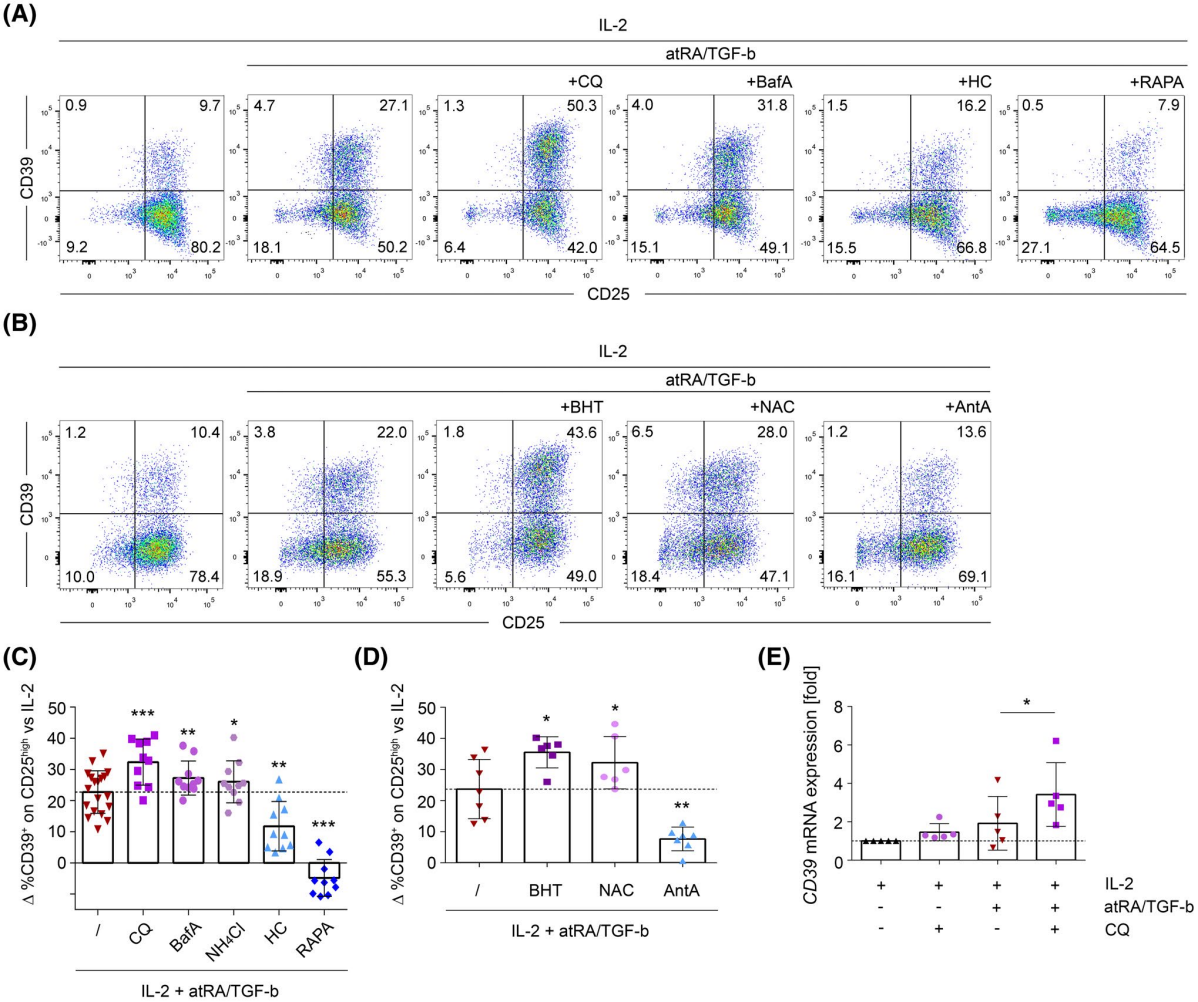
Autophagy and ROS levels modulate CD39 upregulation by atRA/TGF‐b. A, Autophagy inhibitors (chloroquine; CQ, Ammoniumchloride; NH_4_Cl or Bafilomycin A1; BafA) or autophagy inducers (Calcium hydroxycitrate; HC or Rapamycin; RAPA) were added to IL‐2+atRA/TGF‐b‐mediated Treg‐induction before primary stimulation. Cultures with IL‐2 only served as controls. Seven days after Treg‐induction, surface‐expression of CD25 and CD39 was measured. Data show FACS plots of one representative donor. B, ROS‐inhibitors (Butylated hydroxytoluene; BHT or N‐acetyl‐cysteine; NAC) or the mitochondrial ROS‐inducer Antimycin A; AntA were added to IL‐2+atRA/TGF‐b‐mediated Treg‐induction and processed as in (A). C, Statistical analyses of autophagy‐modulators, (D) statistical analysis of ROS‐modulators. C, D, For statistical analyses, CD39 expression values were corrected against values from the control culture. Data are represented as mean ± SD *P ≤ .05; **P < .01; ***P < .001 (paired *t* test). E, After 10 days of IL‐2+atRA/TGF‐b‐mediated Treg‐induction in presence/absence of the autophagy‐inhibitor CQ, CD39 mRNA expression was analyzed by RT‐PCR. Expression rate was calculated using beta‐2‐microglobulin as a reference gene and were set relative to the expression rate in IL‐2 control cells. Data are represented as mean ± SD, *P ≤ .05 (paired *t* test)

Recent studies have linked the process of autophagy to the expression of CD39 on tumor cells.^36^, ^37^ Similarly, the dependence of CD39 expression on mTOR signaling, which also inhibits autophagy,^53^ led us to further investigate a potential role for autophagy in this process. During iTreg induction with atRA/TGF‐b, well‐defined inhibitors of autophagy (chloroquine (CQ), bafilomycin A1 (BafA), and ammonium chloride (NH_4_Cl)) as well as inducers of autophagy (RAPA and hydroxycitrate (HC)) were added to the cultures. We found that autophagy inhibitors uniformly increased the upregulation of CD39 by atRA/TGF‐b, with CQ displaying the most pronounced effect (Figure 3A,C). In accordance, inducers of autophagy significantly counteracted the upregulation of CD39 (Figure 3A,C). Multiple reports have defined that the levels of ROS influence autophagy, with high ROS production resulting in increased autophagy.^54^ In line with the above described observations, the antioxidants NAC and BHT significantly increased induction of CD39 expression. In contrast, the cytochrome c oxidase inhibitor AntA, which increases mitochondrial ROS production^55^, ^56^ strongly downregulated CD39 expression (Figure 3B,D). Increased surface expression of CD39 upon autophagy blockade was associated with increased levels of CD39 mRNA (Figure 3E). Thus, we define that high levels of autophagy counteract the upregulation of CD39 by atRA/TGF‐b via modulation of the gene expression of CD39.

### 3.4 TGF‐b signaling and autophagy do not influence each other in CD4^+^ T‐cells

**FIGURE 4:**
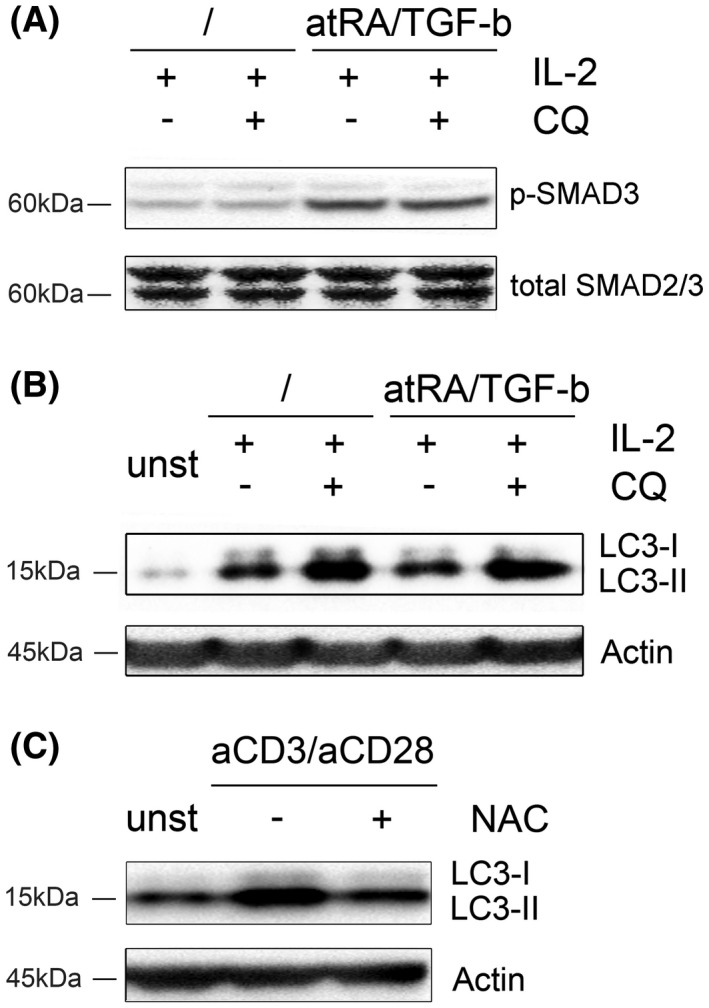
TGF‐b signaling and autophagy do not influence each other in CD4^+^ T‐cells. A, B, 2 × 10^6^ total CD4^+^ T‐cells were preincubated with IL‐2+atRA/TGF‐b in the presence/absence of the autophagy‐inhibitor CQ and were stimulated with anti‐CD3/CD28 coated microbeads. Cells cultured in IL‐2 or IL‐2+CQ only served as controls. After 24 hours, cells were harvested and western blot analyses were performed for (A) phosphorylated SMAD3 and total SMAD2/3 (loading control) and (B) modification of LC3‐I to LC3‐II as indicator for autophagic flux and actin (loading control). C, 2 × 10^6^ total CD4^+^ T‐cells were stimulated with anti‐CD3/CD28 coated microbeads in the presence/absence of the antioxidant NAC. After 24 hours, cells were harvested and western blot analyses were performed for modification of LC3‐I to LC3‐II. Actin was used as loading control. Data show western blots of one representative donor (n = 3)

Subsequently, we investigated whether potential crosstalk between TGF‐b signaling, autophagy, and ROS levels exists. Inhibition of autophagy by CQ did not affect phosphorylation of SMAD3 following exposure to atRA/TGF‐b, indicating that autophagy does not affect canonical TGF‐b signaling (Figure 4A). Furthermore, expression levels of molecules involved in TGF‐b signaling were not affected by this manipulation (Figure S3). Vice versa, we found that atRA/TGF‐b signaling did not influence the autophagy‐dependent conversion of LC3‐I to LC3‐II following activation,^57^ indicating that atRA/TGF‐b signaling does not affect autophagy (Figure 4B). As a confirmation, we also performed analyses using the fluorescent Cyto‐ID dye, which is selectively enriched in autophagic vesicles.^58^ Again, we found no differences between cells activated in IL‐2 or IL‐2 plus atRA/TGF‐b (Figure S4A). In line with the described functions of CQ,^59^ addition to either protocol strongly enhanced accumulation of LC3‐II due to the block in autophagosome turn‐over (Figure 4B). atRA/TGF‐b signaling also did not affect production of ROS in naïve T‐cells following activation (Figure S4B). Reduction of ROS levels by the antioxidant NAC also led to a marked decrease of autophagy confirming the previously described findings also in human T‐cells^60^ (Figure 4C). Thus, no crosstalk between atRA/TGF‐b signaling and ROS‐driven autophagy was detected.

### 3.5 Primary CD39^−^ peripheral blood tTreg show higher levels of ROS and autophagy compared to CD39^+^ tTreg

**FIGURE 5:**
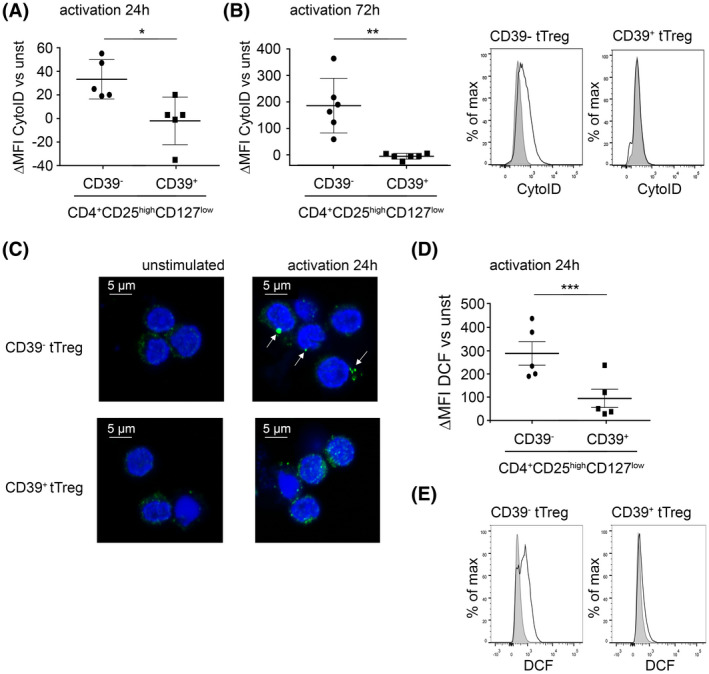
CD39^−^ peripheral blood tTreg show higher levels of ROS and autophagy compared to CD39^+^ tTreg. CD4^+^CD25^high^CD127^low^ tTreg were stringently separated into the CD39^−^ and CD39^+^ subsets using flow cytometric sorting and were activated with anti‐CD3/CD28 coated microbeads for the indicated time points. A, Twenty‐four hours after activation, autophagic vesicles were measured by FACS using Cyto‐ID; MFI of unstimulated cells was subtracted from the MFI of stimulated cells. Data are represented as mean ± SD **P < .01 (paired *t* test). B, Autophagic vesicles were measured 72 hours after activation (see above). Left: statistical analyses (see above), *P ≤ .05 (paired *t* test) Right: Histogram‐overlays of one representative donor showing unstimulated cells (grey histograms) and stimulated cells (black line) of the indicated populations. C, Twenty‐four hours after activation, confocal microscopy for the formation of LC‐3 puncta was performed. High density puncta, indicative for autophagosome formation after activation, are marked with arrows. D, E, Twenty‐four hours after activation, intracellular ROS levels were measured by FACS using DCFDA‐conversion. D, Statistical analyses of intracellular ROS; MFI of unstimulated cells was subtracted from the MFI of stimulated cells. Data are represented as mean ± SD, ***P < .001 (paired *t* test). E, Histogram overlays of one representative donor showing unstimulated cells (grey histogram) and stimulated cells (black line) of the indicated cell populations

Following the results obtained with iTreg, we investigated whether these principles could also be applied for peripheral blood tTreg from healthy individuals. CD4^+^CD25^high^CD127^low^ tTreg were stringently separated into the CD39^−^ and CD39^+^ subsets using flow cytometric sorting. Following activation, CD39^−^ tTreg showed a robust upregulation of autophagy, while CD39^+^ tTreg showed only minimal staining with the Cyto‐ID dye (Figure 5A,B). As a confirmation, we performed confocal microscopy for the formation of LC‐3 puncta in both subsets. In accordance, CD39^−^ tTreg showed high density puncta indicative for autophagosome formation after activation. In contrast, CD39^+^ tTreg showed no high density puncta (Figure 5C). CD39^−^ tTreg also showed a significantly higher ROS production following activation compared to CD39^+^ tTreg (Figure 5D,E). Thus, our results define that CD39^−^ and CD39^+^ tTreg constitute distinct entities also in regard to their capacity to induce autophagy and ROS production following activation.

### 3.6 Manipulation of TGF‐b signaling, autophagy, or ROS levels modulates CD39 expression on peripheral blood tTreg

**FIGURE 6:**
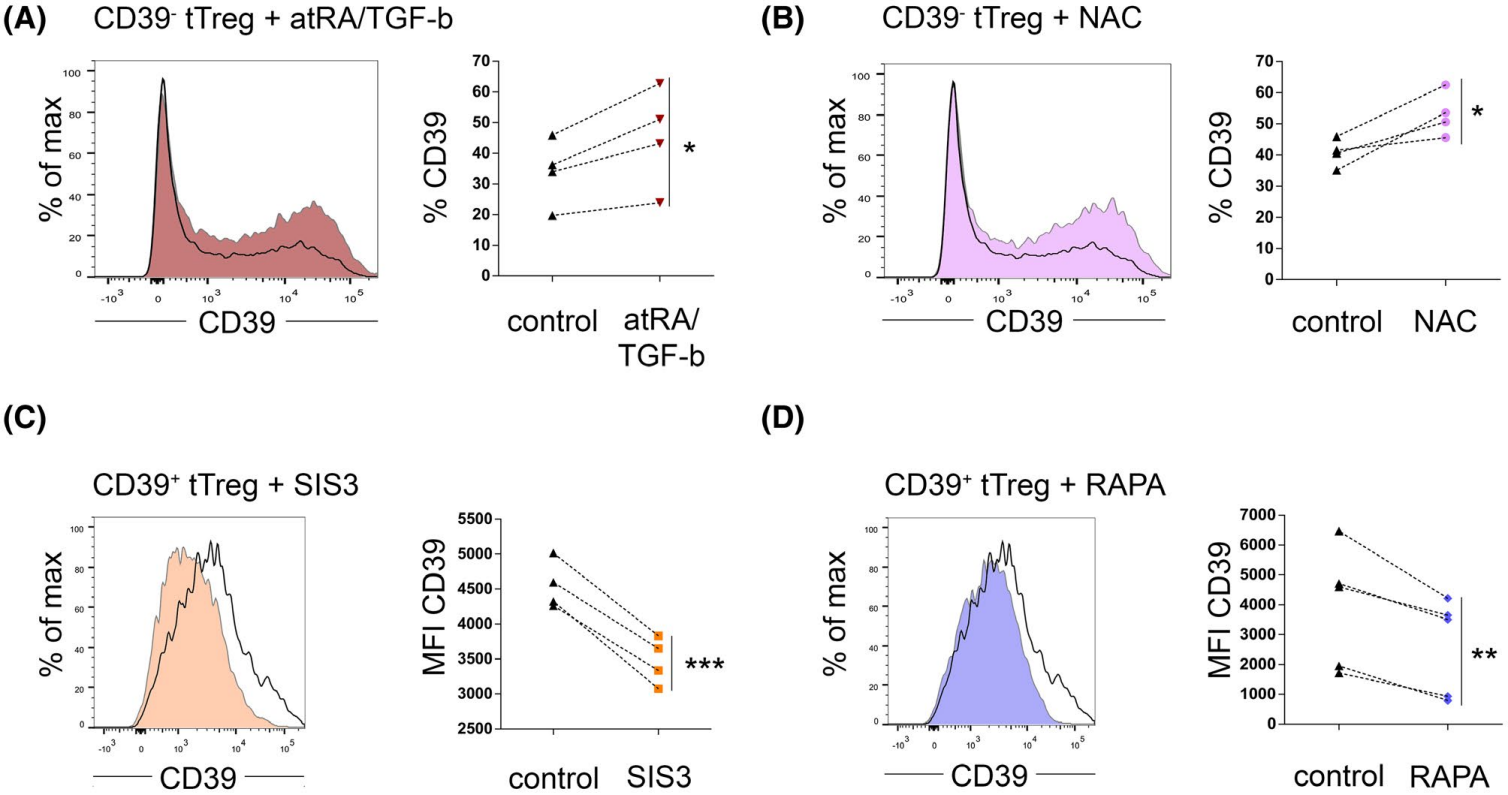
Manipulation of TGF‐b signaling, ROS levels or autophagy modulates CD39 expression on peripheral blood tTreg. A, B, CD4^+^CD25^high^CD127^low^CD39^−^ tTreg were stringently isolated using flow cytometric sorting and were activated with anti‐CD3/CD28 coated microbeads in the presence of (A) atRA/TGF‐b (red) or (B) the antioxidant NAC (pink). Four days after activation, CD39 expression was measured by FACS. Left: Histogram overlays of one representative donor; CD39 expression of tTreg activated without supplements is depicted as black line. Right: Statistical analyses. C, D, CD4^+^CD25^high^CD127^low^CD39^+^ tTreg were stringently isolated using flow cytometric sorting and after 4 days of activation in the presence of (C) the canonical TGF‐b signaling inhibitor of SMAD3 (orange) or (D) the mTOR‐inhibitor RAPA (blue), CD39 expression was measured. Left: histogram overlays of one representative donor; CD39 expression of tTreg activated without supplements is depicted as black line. Right: Statistical analyses. A‐D, Statistical analyses: *P ≤ .05; **P < .01; ***P < .001 (paired *t* test)

In the light of the above described biologic principles of iTreg and CD39^−^ and CD39^+^ tTreg, we assessed whether modulation of TGF‐b signaling, autophagy, or ROS levels could also modulate CD39 levels on tTreg. Following activation, CD39^−^ tTreg upregulated CD39 expression indicating that, similar to naïve T‐cells, CD39 expression is to a certain extent a default process during the activation of tTreg. In accordance with the data obtained from the iTreg polarization experiments, activation in the presence of atRA/TGF‐b significantly increased the upregulation of CD39 expression (Figure 6A). Similarly, inhibition of ROS production by the antioxidant NAC significantly increased the upregulation of CD39 on activated CD39^−^ tTreg (Figure 6B). Vice versa, in CD39^+^ tTreg, blockade of SMAD2/3 signaling by the inhibitor SIS3 and inhibition of mTOR signaling using RAPA significantly downregulated CD39 expression levels (Figure 6C,D).

### 3.7 Genetic disorders of autophagy or ROS production lead to enhanced CD39 expression on tTreg

**FIGURE 7:**
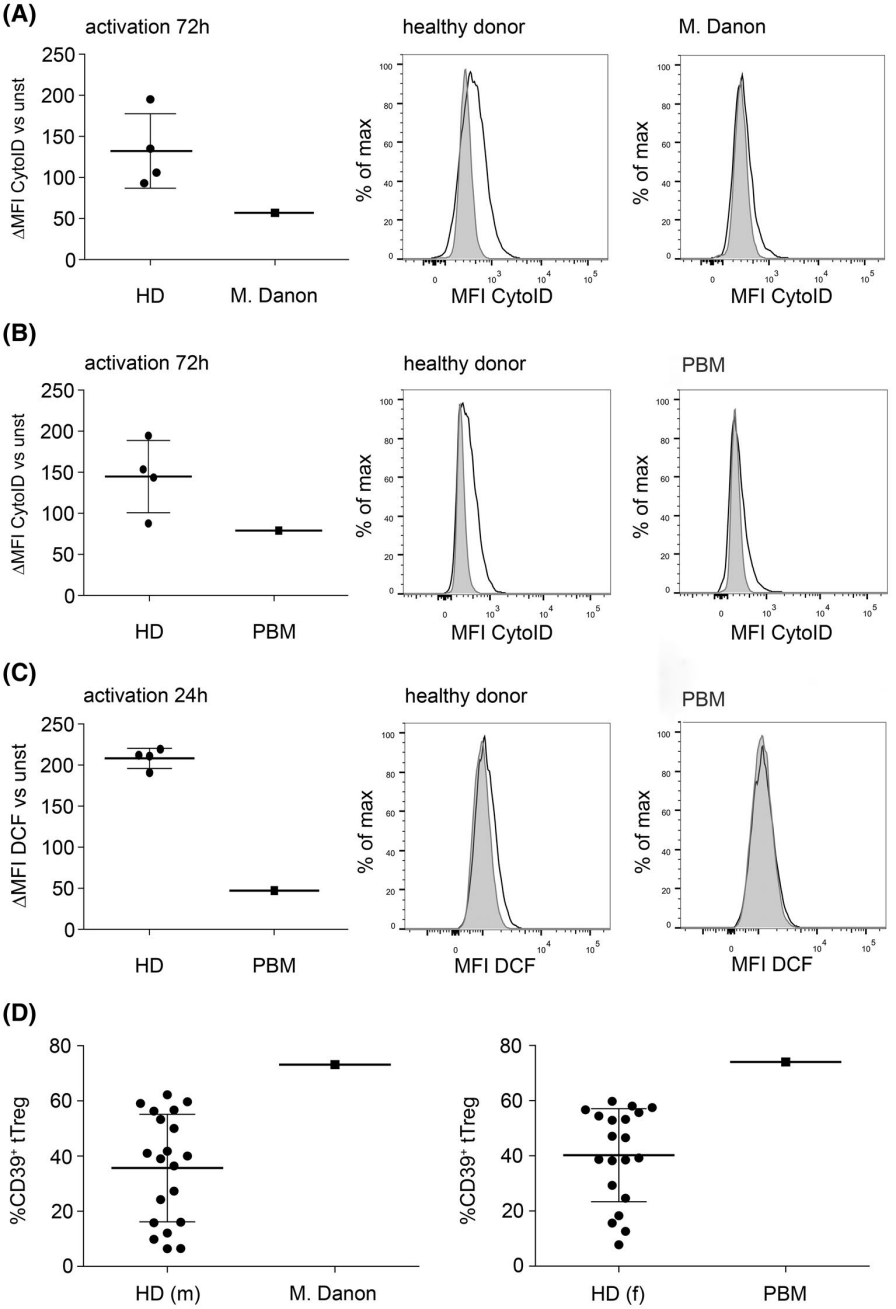
Genetic disorders of autophagy or ROS production lead to enhanced CD39 expression on tTreg. A, CD4^+^ T‐cells from healthy sex‐ and age‐matched donors (HD) as well as from one patient suffering from M. Danon were isolated using flow cytometric sorting and were activated with anti‐CD3/CD28 coated microbeads for 72 hours. Autophagy was measured by FACS using the Cyto‐ID dye. Left: Statistical analyses, MFI of unstimulated cells was subtracted from the MFI of stimulated cells and data are represented as mean ± SD. Right: Histogram overlays of one representative, healthy donor, or a patient suffering from M. Danon showing unstimulated cells (grey histogram) and stimulated cells (black line). B, CD4^+^ T‐cells from a patient suffering from polyglucosan body myopathy (PBM) were processed and measured as above. C, ROS production in CD4^+^ T‐cells of healthy donors or PBM‐patient. Cells were activated for 24 hours and ROS‐levels were measured by FACS using DCFDA‐conversion. D, CD39 expression in peripheral blood CD4^+^CD25^+^CD127^low^ tTreg from healthy sex‐ and age‐matched donors compared to M. Danon (left) or PBM (right), data are represented as mean ± SD

To further assess the relevance of ROS‐driven autophagy for CD39 expression in vivo, we resorted to the analyses of CD4^+^ T‐cells from patients with rare genetic defects leading to impaired autophagy and/or ROS production. We obtained blood samples from a male patient suffering from a defect in the *LAMP‐2* gene, which is causative for Danon's disease and is associated with defects in autophagy.^61^ Similarly, we also analyzed CD4^+^ T‐cells from a recently described female patient suffering from polyglucosan body myopathy (PBM) due to a mutation in the *RBCK‐1* gene.^38^ Following activation, CD4^+^ T‐cells from both patients showed low levels of autophagy in comparison to sex‐ and age‐matched control donors (Figure 7A,B). Furthermore, ROS production was absent in the cells from the PBM patient (Figure 7C). Phenotyping of peripheral blood tTreg from both patients revealed that the percentage of CD39^+^ cells within this population was strongly increased in comparison to sex‐ and age‐matched donor populations (Figure 7D). Importantly, neither surface expression of CD25 and CD127 nor intracellular expression of FOXP3 was affected in these patients, suggesting that the genetic defects did not alter the overall phenotype of their tTreg subsets (Figure S5).

### 3.8 Transcriptomic analyses of CD39^−^/CD39^+^ tTreg identify SOX4 as key transcription factor in the regulation of CD39 expression

**FIGURE 8:**
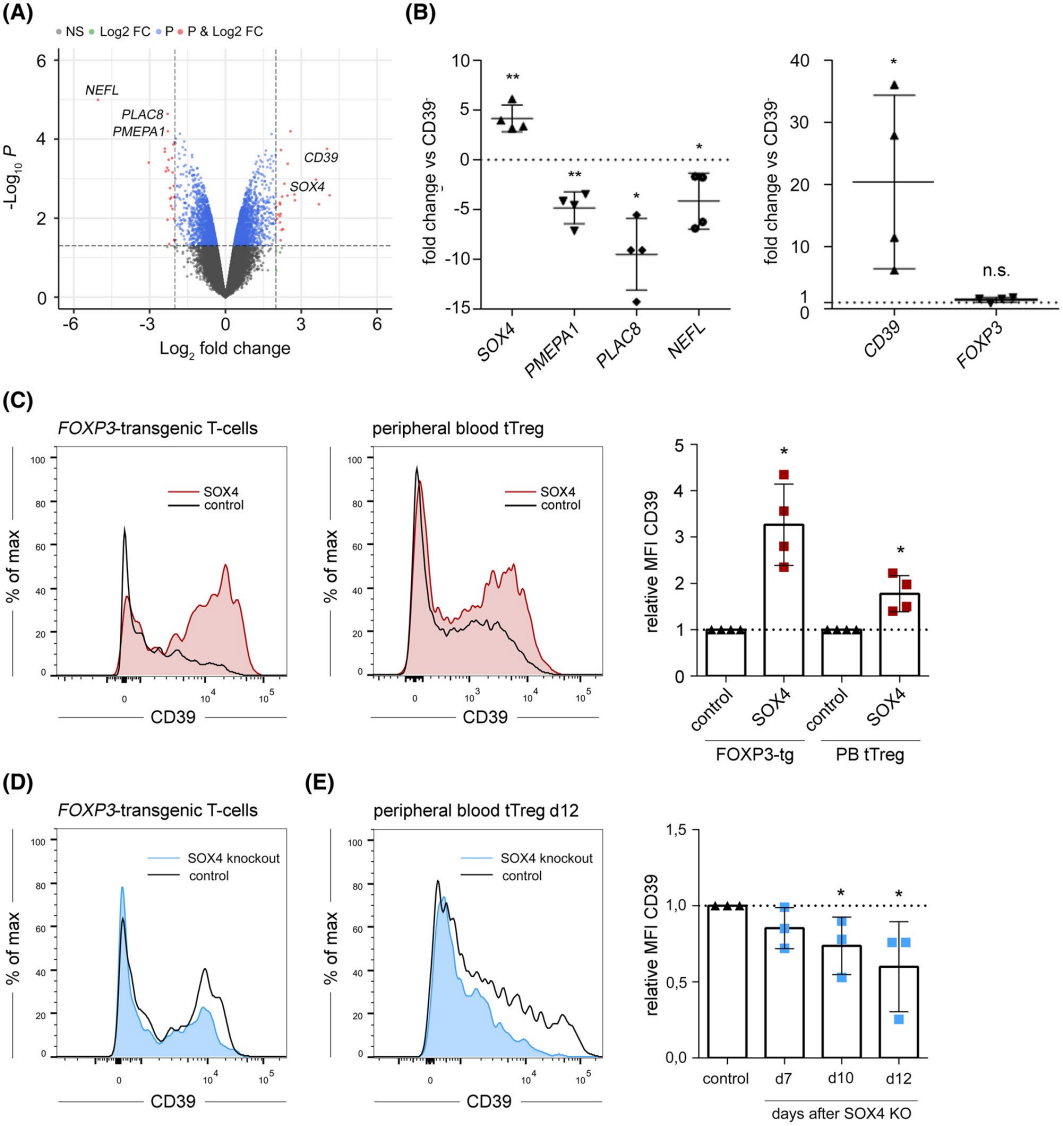
Transcriptomic analyses of CD39^−^ and CD39^+^ tTreg identify SOX4 as key transcription factor in the regulation of CD39 expression. A, Volcano‐blot; CD4^+^CD25^high^CD127^low^CD39^−/+^ tTreg were FACS‐sorted and microarray analyses were performed (n = 3). Red data points outside the black dashed lines indicate significant regulation on CD39^+^ tTreg in comparison to CD39^−^ tTreg. B, mRNA expression of the indicated genes in CD39^−/+^ tTreg was analyzed by RT‐PCR. Expression rates were calculated using *GAPDH* as a reference gene and fold change of mRNA‐expression in CD39^+^ tTreg compared to CD39^−^ tTreg was calculated. Data are shown as mean ± SD, *P ≤ .05; **P < .01 (paired *t* test). C, CD39‐expression on CD4^+^ *FOXP3*‐transgenic (tg) T‐cells (left histogram) or peripheral blood (PB) tTreg (right histogram) following retroviral transduction with the transcription factor *SOX4* using the pMMP‐IRES‐GFP vector. The black line indicates empty vector transduced cells (control), red histograms represent *SOX4*‐transduced cells. Right: Statistical analyses; data are represented as mean ± SD *P < .05 (paired *t* test). D, CD39‐expression on CD4^+^ *FOXP3*‐tg T‐cells following Crispr/Cas9 mediated knockout (KO) of *SOX4.* The black line indicates control‐KO cells, blue histograms represent *SOX4*‐KO cells. The histogram shows one representative donor. E, CD39‐expression on PB tTreg following Crispr/Cas9 mediated knockout of *SOX4*. Left: Histogram of one representative donor. Right: Statistical analyses, data are represented as mean ± SD

In order to further define molecular mechanisms underlying the distinct features of CD39^−^ and CD39^+^ tTreg, we performed microarray analyses of stringently FACS‐sorted tTreg populations (GEO database #GSE131743). Genes displaying either significantly increased or decreased expression in the CD39^+^ tTreg population were screened for involvement in TGF‐b signaling or autophagy. We identified strong downregulation of the TGF‐b signaling inhibitor *PMEPA1* ^62^ and significant upregulation of the TGF‐b downstream transcription factor *SOX4*.^63^ Furthermore, two genes with putative involvement in autophagy, *NEFL* ^64^ and *PLAC8* ^65^ were strongly downregulated in CD39^+^ tTreg (Figure 8A). This expression pattern was confirmed by RT‐PCR from specimen of four further healthy individuals (Figure 8B). Retroviral transduction of *SOX4* significantly increased CD39 surface expression both in *FOXP3*‐transgenic T‐cells as well as in FACS‐sorted peripheral blood tTreg (Figure 8C). For further validation, we performed Crispr/Cas9 mediated knockout of *SOX4* in *FOXP3*‐transgenic T‐cells as well as in FACS‐sorted peripheral blood tTreg following the protocol established by Roth et al.^49^ In accordance to the results presented above, knockout of *SOX4* significantly reduced CD39 expression in FOXP3‐transgenic T‐cells (Figure 8D) as well as peripheral blood tTreg (Figure 8E,F). Thus, these data reveal a direct role for SOX4 in the regulation of CD39 expression in human peripheral blood tTreg.

## 4 DISCUSSION

The ATP‐degrading ectonucleotidase CD39 plays an important role in the regulation of immune responses under physiological conditions. Additionally, aberrant CD39 expression has been described for autoimmune diseases.^13^, ^21^ In recent years, the relevance of CD39 as mechanism of tumor immune evasion in various cancers has been demonstrated^29^, ^37^ and first preclinical studies with CD39 inhibitors have shown promising results.^28^, ^66^, ^67^, ^68^ CD39 expression has been defined as an important mechanism of suppression on murine and human tTreg.^12^, ^13^, ^69^ In this context, a clear‐cut model was established with CD39 and CD73 being expressed on the majority of murine tTreg and CD39 expression being dependent on the transcription factor Foxp3.^13^ In contrast, first studies by Rissiek et al, which were confirmed in this study, provided evidence that CD39 shows a highly heterogeneic expression on tTreg in humans, indicating that factors other than FOXP3 might govern its expression.^18^ Besides FOXP3, the transcription factor HELIOS is a major determinant of Treg features.^8^ However, in phenotypic analyses of peripheral blood tTreg we found no correlation between the expression of FOXP3, HELIOS, and CD39. Furthermore, genetic overexpression of FOXP3 and HELIOS in human CD4^+^ T‐cells did not affect CD39 expression. Thus, we here provide first formal proof that CD39 expression on CD4^+^ T‐cells and in particular on Treg is independent of the key transcription factors FOXP3 and HELIOS. Thus, our data further extend the knowledge about FOXP3‐dependent and ‐independent mechanisms in tTreg.^6^

Given these observations, we aimed to define mechanisms or transcription factors other than the above described “canonical” tTreg transcription factors for their involvement in the gene regulation of CD39. As a model system, we first used the in vitro polarization of naïve T‐cells into iTreg. Two major protocols have been described in this respect which both target distinct molecular signaling events, one using culture in atRA/TGF‐b^2^ and one using the mTOR inhibitor RAPA.^3^ Although both protocols have been well established, an in‐depth analysis and comparison regarding the fine‐specificity of the generated iTreg has not been performed so far. Our experiments confirm that both protocols are capable to generate iTregs which show robust expression of CD25 and suppressive capacity in coculture experiments. In stark contrast, CD39 expression on these differentially generated iTreg showed an opposing pattern, with atRA/TGF‐b inducing high levels on iTreg and RAPA iTreg displaying low levels. In our experiments, we also found that both naïve T‐cells as well as tTreg de novo expressed intermediate levels of CD39 upon TCR/CD28‐triggering. This indicates that CD39 expression is a default process after T‐cell activation which is strongly superinduced by canonical TGF‐b signaling typically found in a tolerogenic milieu. Addition of RAPA overrules both basal as well as TGF‐b‐induced upregulation of CD39, highlighting that mTOR signaling and associated mechanisms play an indispensable role for this process.

The mTOR kinase complex is involved in various activation‐induced processes in T‐cells including protein translation, cell survival, proliferation, and regulation of metabolic features.^70^, ^71^ One particular feature is the inhibition of macro‐autophagy (commonly referred to as autophagy).^53^ We demonstrate that modulation of autophagy during atRA/TGF‐b‐mediated Treg‐induction strongly influenced CD39 expression. In our experiments inhibition of autophagy led to increased CD39 expression, which was due to enhanced translation of the *CD39* gene. Along those lines, autophagy inducers showed the opposing effect, indicating that the autophagic state of the T‐cells regulates CD39 expression in both directions. Preceding studies have established possible links between the regulation of autophagy and the expression of CD39 on tumor cells.^36^, ^37^ In this light, our data provide evidence that CD39 regulation by autophagy is not only a tumor‐cell‐specific mechanism, but also occurs in lymphoid cells thus shaping their function.

Multiple signals direct the process of autophagy in different cell types. Recent studies have given distinct proof that the levels of ROS are closely linked to the autophagic state of diverse cells, with high ROS levels supporting high autophagic flux.^54^ We were able to establish, that ROS production also affects autophagy in human CD4^+^ T‐cells. Accordingly, downregulation of ROS levels by antioxidants induced increased CD39 expression similar to autophagy inhibitors, while ROS inducers had the opposing effects. Of note, these observations in CD4^+^ T‐cells are in contrast to the situation in CD8^+^ T‐cells, which was recently established by Bai et al In this study, the authors show that CD39 expression on human CD8^+^ T‐cells is upregulated by high ROS levels.^72^ Taken together, these findings indicate that human CD4^+^ and CD8^+^ T‐cells differently integrate metabolic cues for distinct cellular functions, a notion that has been investigated by us in more detail recently.^73^ Mechanistically, our observations also show that TGF‐b signaling and ROS‐driven autophagy do not influence each other but rather act in synergy on the expression of CD39. Of note, inhibition of autophagy in control cultures without TGF‐b only minimally affected CD39 levels. These findings show that autophagy selectively modulates the effects of TGF‐b on CD39 expression. Also in this respect it remains to be determined, whether other TGF‐beta driven genes are similarly influenced by modulation of autophagy. Importantly, manipulation of TGF‐b signaling, ROS production, and mTOR signaling affected the expression of CD39 on tTreg to a similar degree as found in the iTreg experiments thus validating their relevance.

The above described mechanisms did not affect expression of the ectonucleotidase CD73, which cooperates with CD39 in the conversion of ATP to adenosine. These data support the hypothesis, that CD39 and CD73 expression are differentially regulated by distinct molecular processes in human T‐cells. This is further supported by the strictly segregated expression pattern of CD39 and CD73 on peripheral blood T‐cell subsets. Of note, TGF‐b was shown to promote upregulation of CD73 on murine CD4^+^ T‐cells while leaving CD39 levels unaffected.^74^ These findings again point out that the CD39/CD73 machinery is differentially regulated between human and murine T‐cells. Consequently, further studies are clearly warranted to obtain a better understanding about the validity of murine models on CD39/CD73 gene regulation for potential translation into human therapeutic settings.

The insights gained into molecular processes using iTreg polarization as model system were validated with regard to peripheral blood tTreg. We found that CD39^−^ tTreg robustly induced both ROS production as well as autophagy following anti‐CD3/anti‐CD28 activation. In stark contrast, CD39^+^ T‐cells showed no induction of these processes following activation. Thus, these two subsets do not simply represent phenotypic variations from the same cellular entity, but display differential metabolic features. The general importance for autophagy in the regulation of CD39 expression in tTreg is supported by the situation in patients with genetic defects resulting in low autophagy. We found that in a patient suffering from M. Danon and in a patient with polyglucosan body myopathy, accumulation of autophagic vesicles was nearly absent in CD4^+^ T‐cells, indicating that autophagy is not induced in these cells upon activation in contrast to healthy controls. In both patients CD39 expression on peripheral blood tTreg surpassed levels found in a corresponding healthy donor population, while levels of other Treg‐associated markers such as FOXP3 and CD25 were not altered. These observations thus validate the correlations established by us in vitro in a human in vivo setting and confirm the selectivity for CD39 expression. To our knowledge, this is also the first description about dysregulation of ROS production and autophagy in cells of a patient suffering from polyglucosan body myopathy. In this context, it remains to be determined whether these functional consequences are directly caused by the underlying genetic defect in the *RBCK‐1* gene or whether they are secondary to the metabolic disturbances caused by this mutation.

Subsequent transcriptomic analyses revealed that the distinct biological properties of CD39^−^/CD39^+^ tTreg are maintained by specific underlying gene expression profiles. Two genes which showed the strongest downregulation in CD39^+^ tTreg were neurofilament light chain (*NEFL*) and Placenta Associated 8 (*PLAC8*). For both factors, putative roles in autophagy have been proposed. NEFL has been discussed in the initiation of autophagy,^64^ while PLAC8 mediates autophagosome/autolysosome fusion.^65^ Thus, our findings imply a model in which autophagy in CD39^+^ tTreg is actively suppressed by the downregulation of genes involved in different stages of this process. From these observations several key questions arise. First, the role of *NEFL* and *PLAC8* in autophagy, especially in T‐cells, has not been fully defined. Second, the activity of most factors classically involved in autophagy (eg, ATG proteins) is not regulated at the transcriptional level but rather through protein stability or posttranslational modifications.^75^ In this respect, further studies about the components and regulation of autophagy in CD39^−^/CD39^+^ human tTreg are clearly warranted. It also remains to be assessed which mechanisms in the tumor microenvironment influence the autophagic milieu, thus promoting CD39 expression on tumor‐infiltrating tTreg and peripherally‐induced Treg.

The importance of TGF‐b signaling for CD39 expression was also reflected in the differential gene expression between CD39^−^/CD39^+^ tTreg. CD39^−^ tTreg showed highly increased levels of *PMEPA1*, which has been described as molecular inhibitor of canonical TGF‐b signaling via SMAD3.^62^, ^76^ Thus, CD39^−^ tTreg actively suppress TGF‐b signaling thereby inhibiting CD39 upregulation. The role of TGF‐b is further supported by the finding that expression of the TGF‐b downstream transcription factor *SOX4* ^63^ is strongly upregulated in CD39^+^ tTreg. Retroviral overexpression of *SOX4* was sufficient to increase CD39 expression levels both in FOXP3‐transgenic as well as peripheral blood tTreg, while Crispr/Cas9‐mediated knockout of *SOX4* significantly reduced CD39 expression on peripheral blood tTreg. Thus, we define SOX4 as key regulatory factor for CD39 expression. So far, SOX4 has mainly been described in developmental biology^77^, ^78^ and also in the context of tumor differentiation.^79^, ^80^ In this study, we established a novel crucial role for SOX4 as central transcription factor in immune tolerance.

In conclusion, we describe novel biological principles specifically involved in the regulation of distinct features of T‐cell tolerance. During the polarization of naïve T‐cells into Treg, atRA/TGF‐b upregulate CD39 expression in an mTOR dependent manner, which is counteracted by ROS‐driven autophagy. Similarly, CD39^+^ tTreg maintain their status by constitutive autocrine/paracrine TGF‐b signaling resulting in high SOX4 levels, which directly enhance CD39 expression. This status is further supported by the downregulation of ROS production and the lack of autophagy. On the contrary, CD39^−^ tTreg express inhibitors of canonical TGF‐b signaling and are able to mount ROS production and autophagy during activation, thereby stabilizing their CD39^−^ status. Thus, we characterize CD39^−^/CD39^+^ tTreg as distinct subsets defined by the differential expression of the transcription factor SOX4 and specific metabolic features.

## CONFLICT OF INTEREST

All authors declare no competing interests.

## AUTHOR CONTRIBUTIONS

M.C. Gerner performed cloning of transgenes, preparation of cell samples, flow cytometric, and biochemical analyses, L.S. Ziegler and R.L.J. Schmidt contributed to cell isolation, cell culture, and flow cytometric analyses, M. Krenn and F. Zimprich contributed clinical data and blood specimen from the PBM patient, K. Uyanik‐Ünal, V. Konstantopoulou, and K. Boztug contributed clinical data and blood specimen from the M. Danon patient, S. Derdak provided bioinformatics analyses of the transcriptomics experiments, G. Del Favero provided confocal microscopy data, I. Schwarzinger provided support with flow cytometric analyses and cell sorting, K.G. Schmetterer coordinated the project and wrote the manuscript together with M.C. Gerner. All authors critically read the manuscript and contributed to the final formulation.

## Supporting information

Supplementary Material
