# Supplementary material for: The TGF‐b/SOX4 axis and ROS‐driven autophagy co‐mediate CD39 expression in regulatory T‐cells
Source: FASEB J. 2020 Apr 22;34(6):8367–84. doi: 10.1096/fj.201902664 (PMC7317981; doi:10.1096/fj.201902664)
Supplement: Supplementary file 1 — Supplementary Material [file FSB2-34-8367-s001.docx]

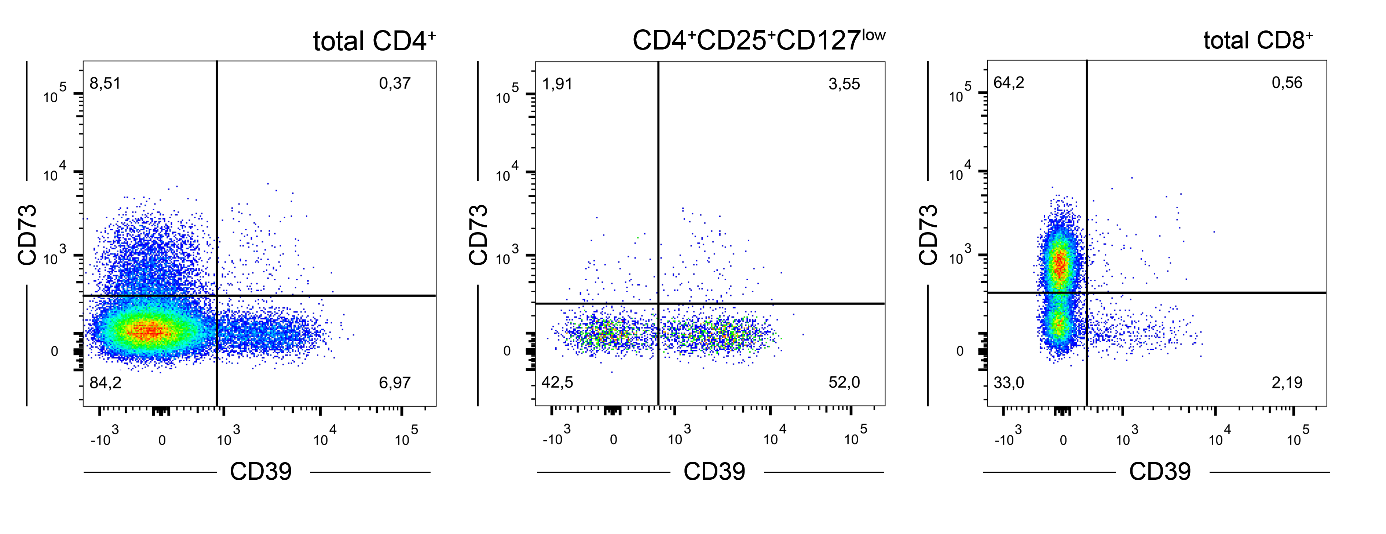


**Supplemental Figure S1.** Expression of CD39 and CD73 on different T-cell subsets. Representative FACS-plots of CD39 and CD73 surface-expression on peripheral blood CD4^+^ T-cells (left and central) as well as CD8^+^ T-cells (right) from one healthy donor (n=20).


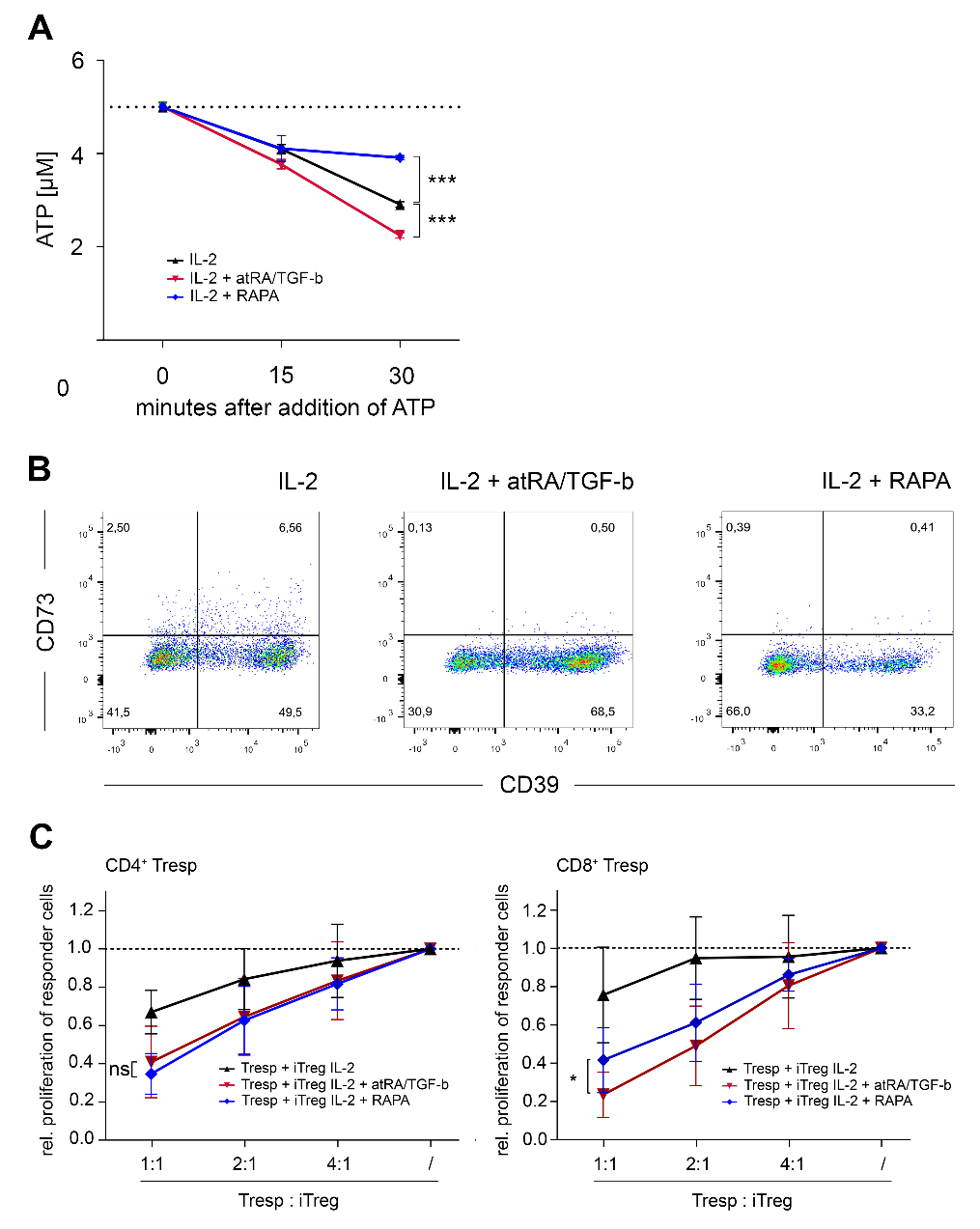


**Supplemental Figure S2.** Suppressive capacity of iTreg. For iTreg induction, naïve CD4^+^CD25^-^CD39^-^CD45RO^-^ cells were preincubated with IL-2 in combination with either atRA/TGF-b or RAPA and cells were stimulated with anti-CD3/CD28 coated microbeads. Cultures with IL-2 only served as controls. After 14 days of Treg-induction, the CD25^high^ population of the respective iTreg were isolated by FACS-sorting A) 7x10^4^ cells were cultured with 5µM ATP, after 15 min and 30 min, supernatants were collected and ATP-concentration was measured using a luciferase-based ATP-detection assay. Data represent three replicates of one representative experiment (n=5), data are represented as mean±SD ***P<0.001 (one-way ANOVA). B) Surface-expression of CD39 and CD73 was measured by FACS. Data show representative FACS-plots from one healthy donor after 14d of Treg-induction (n=5). C) Suppressive potential of iTregs: iTregs were co-cultured with 7x10^4^ autologous cell proliferation-dye (CPD)-labeled CD4^+^ or CD8^+^ Tresp in different ratios. Four days after stimulation with anti-CD3/CD28 coated microbeads, the proliferation rate of Tresp was measured by FACS. Data are represented as proliferation index relative to the control culture without iTreg and are shown as mean±SD, CD4^+^ Tresp n=11, CD8^+^ Tresp n=8, *P≤0.05; not significant (ns) P>0.05 (paired t-test).


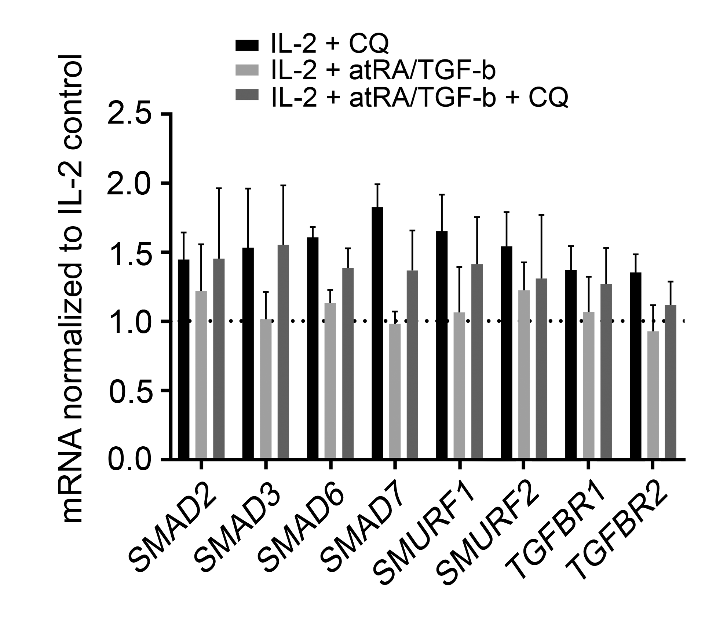


**Supplemental Figure S3.** Inhibition of autophagy does not affect expression levels of molecules involved in TGF-b signaling. 24 hours after IL2+atRA/TGFb-mediated Treg-induction in the presence/absence of the autophagy-inhibitor CQ, expression of the indicated mRNAs involved in TGF-b signaling was analyzed by RT-PCR. Expression rate was calculated using GAPDH as a reference gene and were set relative to the expression rate in IL2-control cells. Data are represented as mean±SD (n=3). No statistically significant differences between different specimen were found.


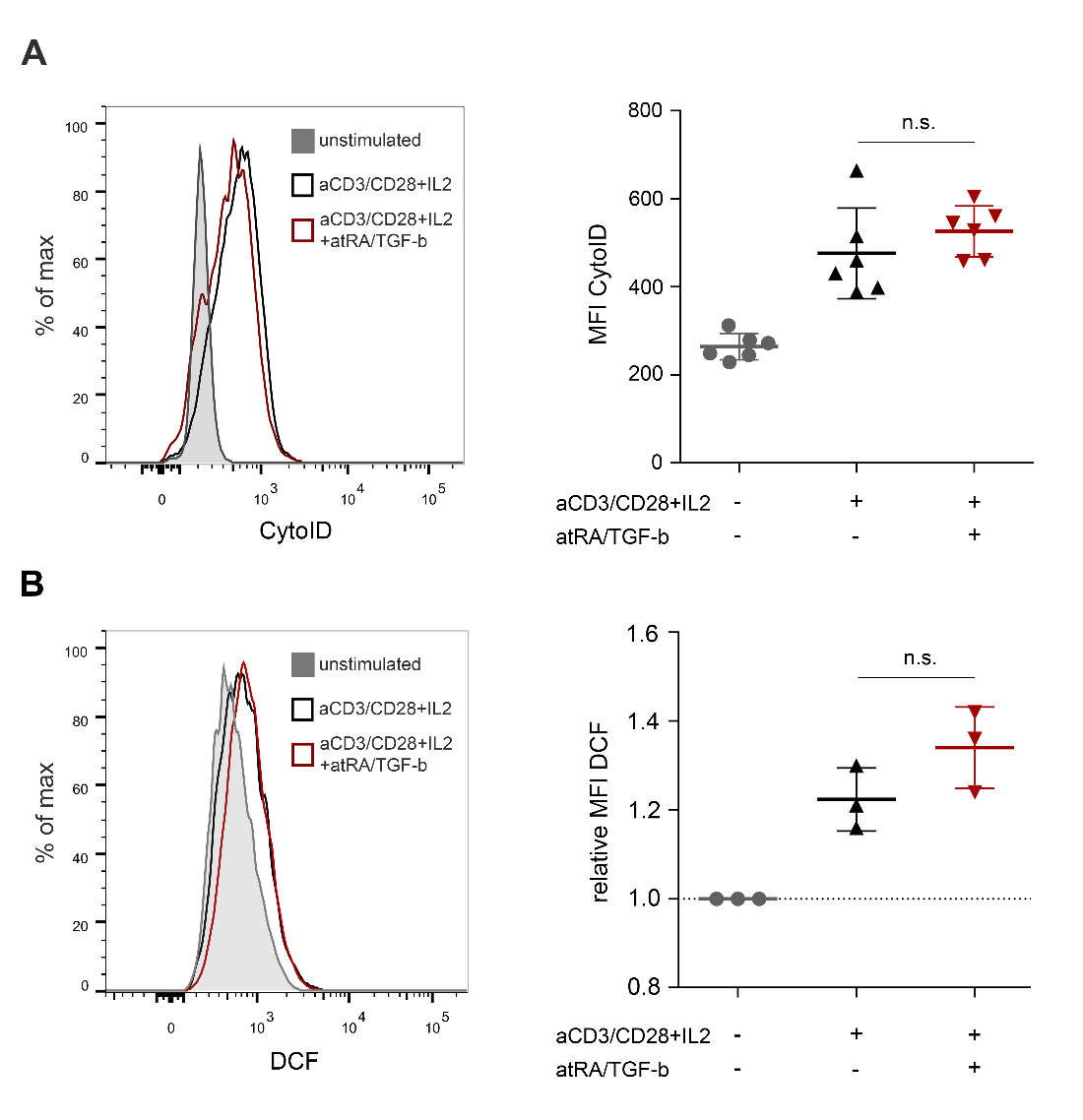


**Supplemental Figure S4.** TGF-b signaling and ROS-driven autophagy do not interact in CD4^+^ T-cells. A, B) 1x10^5^ naïve CD4^+^CD25^-^CD39^-^CD45RO^-^ cells were preincubated with IL2+atRA/TGF-b and were stimulated with anti-CD3/CD28 coated microbeads. A) After 72h in culture, autophagic flux was measured by FACS using the CytoID dye, which selectively stains autophagic vesicles. Data are represented as mean±SD; not significant (n.s.) P>0.05 (paired t-test). B) After 24h, intracellular ROS-levels were measured by the conversion of DCFDA to DCF using flow cytometry. The MFI of DCF fluorescence of activated cells was set relative to unstimulated cells. Data are represented as mean±SD, not significant (n.s.) P>0.05 (paired t-test).


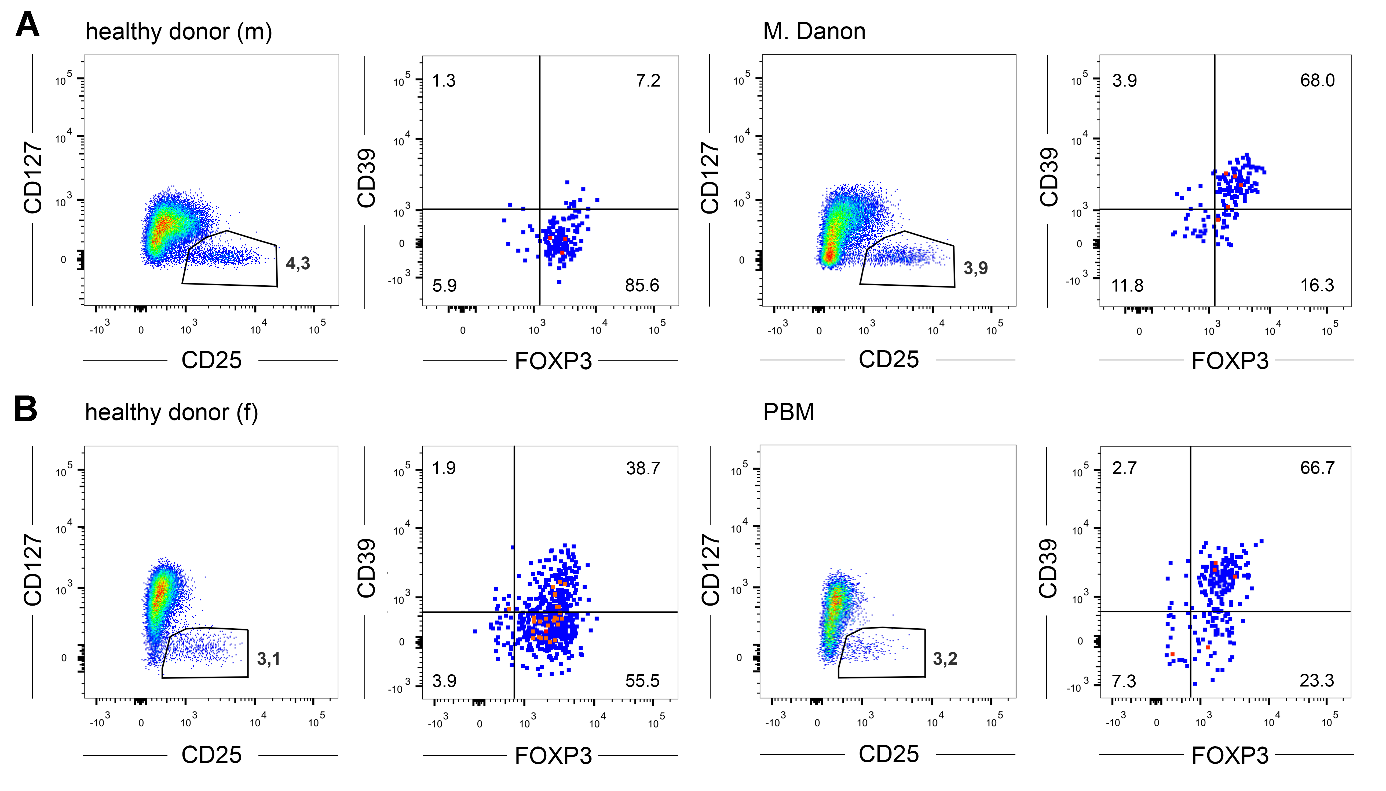


**Supplemental Figure S5.** The phenotype of tTreg is not altered in patients with M.Danon or PBM. A, B) FACS-plots of the tTreg-marker CD25, CD127 (both surface) and FOXP3 (intracellular) in peripheral blood CD4^+^ T-cells. CD39 and FOXP3 expression on cells gated according to the CD3^+^CD4^+^CD25^high^CD127^low^ phenotype is shown. A) Left: FACS-plots of one representative male age-matched healthy donor. Right: FACS-plots of one Morbus Danon patient. B) Left: FACS-plots of one representative female age-matched healthy donor. Right: FACS-plots from one polyglucosan body myopathy (PBM) patient.

| **Target** | **Sequence 5´ 🡪 3´** |
| --- | --- |
| b2m | Forward: GGA ATT GAT TTG GGA GAG CAT C  Reverse: CAG GTC CTG GCT CTA CAA TTT ACT AA |
| CD39 | Forward: GGG AGC ACA TCC ATT TCA TT  Reverse: TCA TGT TGG TCA GGT TCA GC |
| FOXP3 | Forward: AAC ATG CGA CCC CCT TTC  Reverse: ATT GAG TGT CCG CTG CTT CT |
| GAPDH | Forward: CGA GCC ACA TCG CTC AGA CA  Reverse: GGC GCC CAA TAC GAC CAA AT |
| NEFL | Forward: TCC TCA ACG TGA AGA TGG CT  Reverse: TGG TGA AAC TGA GTC GGG TC |
| PMEPA1 | Forward: GGA GGA GAG AAG ATG CCC TG  Reverse: CTC TGG GAT TCC GTT GCC TG |
| SMAD2 | Forward: TGC ACC ATA AGA ATG AGT TTT G  Reverse: TGA AGT TCA ATC CAG CAA GG |
| SMAD3 | Forward: TAC CAG TTG ACC CGA ATG TG  Reverse: AGC AGG GGG TAC TGG TCA C |
| SMAD6 | Forward: TGT CCG ATT CCA CAT TGT CT  Reverse: ACA TGC TGG CGT CTG AGA AT |
| SMAD7 | Forward: CAG ATG CTG TGC CTT CCT C  Reverse: CAG GCT CCA GAA GAA GTT GG |
| SMURF1 | Forward: CCT GAT AGA CGC GAA CAC AG  Reverse: GCT GTC AGC AGC TTC TCG TA |
| SMURF2 | Forward: CCA GAT TGA TGC CTG CAC TA  Reverse: AGC TTT CAT AGG GTG GAA GTG C |
| SOX4 | Forward: TTT TAA CTT CGA GCC CGG CT  Reverse: AGC CAG TCT CCC GAG ATC AT |
| TGFBR1 | Forward: GTC ATG AAA ACA TCC TGG GAT TT  Reverse: ATC TGA CAC CAA CCA GAG CTG |
| TGFBR2 | Forward: GTC TGT GGA TGA CCT GGC TA  Reverse: TCA TCC TGG ATT CTA GGA CTT CTG |

**Supplemental Table S1. Sequences of primers used for RT-PCR.**

| **SOX4 crRNA sequences 5´ 🡪 3´** | | |
| --- | --- | --- |
| Sequence 1 | TGGTGTGGTCGCAGATCGAG |  |
| Sequence 2 | CGAGAACACGGAAGCGCTGC |  |
| Sequence 3 | GGTCGCTTGATGTGCCCACT |  |
| Sequence 4 | GATCTGCGACCACACCATGA |  |
| Sequence 5 | GCTGGTGCAAGACCCCGAGT |  |
| Sequence 6 | ACGACCCGAGCTGGTGCAAG |  |
| **SOX4 sequencing primers 5´ 🡪 3´** | |  |
| Forward | ACTCTCCAGCCTGGGAACTATAA |  |
| Reverse | GGACTTCACCTTCTTCCTGGG |  |

**Supplemental Table S2. RNA sequences and sequencing primer used for Crispr/Cas9-mediated SOX4 knockout.**
